# RNA-seq analysis of *virR* and *revR* mutants of *Clostridium perfringens*

**DOI:** 10.1186/s12864-016-2706-2

**Published:** 2016-05-23

**Authors:** Lee-Yean Low, Paul F. Harrison, Ya-Hsun Lin, John D. Boyce, Julian I. Rood, Jackie K. Cheung

**Affiliations:** Infection and Immunity Program, Biomedicine Discovery Institute and Department of Microbiology, Monash University, Clayton, 3800 Australia; Monash Bioinformatics Platform, Monash University, Clayton, 3800 Australia

**Keywords:** RNA-seq, Gene transcription, Reciprocal gene expression, *C. perfringens*

## Abstract

**Background:**

*Clostridium perfringens* causes toxin-mediated diseases, including gas gangrene (clostridial myonecrosis) and food poisoning in humans. The production of the toxins implicated in gas gangrene, α-toxin and perfringolysin O, is regulated by the VirSR two-component regulatory system. In addition, RevR, an orphan response regulator, has been shown to affect virulence in the mouse myonecrosis model. RevR positively regulates the expression of genes that encode hydrolytic enzymes, including hyaluronidases and sialidases.

**Results:**

To further characterize the VirSR and RevR regulatory networks, comparative transcriptomic analysis was carried out with strand-specific RNA-seq on *C. perfringens* strain JIR325 and its isogenic *virR* and *revR* regulatory mutants. Using the edgeR analysis package, 206 genes in the *virR* mutant and 67 genes in the *revR* mutant were found to be differentially expressed. Comparative analysis revealed that VirR acts as a global negative regulator, whilst RevR acts as a global positive regulator. Therefore, about 95 % of the differentially expressed genes were up-regulated in the *virR* mutant, whereas 81 % of the differentially expressed genes were down-regulated in the *revR* mutant. Importantly, we identified 23 genes that were regulated by both VirR and RevR, 18 of these genes, which included the sporulation-specific *spoIVA*, *sigG* and *sigF* genes, were regulated positively and negatively by RevR and VirR, respectively. Furthermore, analysis of the mapped RNA-seq reads visualized as depth of coverage plots showed that there were 93 previously unannotated transcripts in intergenic regions. These transcripts potentially encode small RNA molecules.

**Conclusion:**

In conclusion, using strand-specific RNA-seq analysis, this study has identified differentially expressed chromosomal and pCP13 native plasmid-encoded genes, antisense transcripts, and transcripts within intergenic regions that are controlled by the VirSR or RevR regulatory systems.

**Electronic supplementary material:**

The online version of this article (doi:10.1186/s12864-016-2706-2) contains supplementary material, which is available to authorized users.

## Background

*Clostridium perfringens* is a Gram-positive, spore-forming anaerobic rod that is ubiquitous in the environment and is found in the gastrointestinal tract of humans and animals [1, 2]. It causes a wide range of diseases in humans, such as clostridial myonecrosis or gas gangrene, food poisoning and necrotizing enterocolitis. In animals it causes lamb dysentery, ovine enterotoxemia and avian necrotic enteritis [1, 3]. These diseases are primarily toxin-mediated and the toxin genes can be either chromosomal or plasmid-encoded [4]. For example, in gas gangrene strains, the major toxin genes *plc* (α-toxin) and *pfoA* (perfringolysin O or θ-toxin) are chromosomally encoded, whilst in animal isolates, the β-toxin, ε-toxin, ι-toxin and NetB toxin genes are present on plasmids [4].

Of the 20 known toxins or hydrolytic enzymes produced by *C. perfringens* [2, 5, 6], α-toxin and perfringolysin O are the primary virulence factors involved in the pathogenesis of gas gangrene [7, 8]. Extracellular hydrolytic enzymes such as sialidases (NanI and NanJ) have also been implicated in disease and postulated to modulate the effect of α-toxin [9]. Furthermore, these enzymes have been proposed to act as spreading factors that destroy the physical properties of tissue matrices and intercellular spaces, thereby aiding in the spread of bacteria within the host [10]. However, mutations in the *nanI* and *nanJ* genes did not affect virulence in the mouse myonecrosis model [11].

The production of α-toxin, perfringolysin O and some extracellular enzymes is regulated by the VirSR two-component system [12, 13], small regulatory RNA molecules (sRNAs) [13, 14], and the RevR orphan response regulator [15]. The VirSR regulatory system is a typical two-component signal transduction system consisting of the membrane-bound sensor histidine kinase, VirS, and the cytoplasmic response regulator, VirR [12, 16]. In response to an appropriate quorum sensing signal, which has been proposed to be an Agr-like autoinducing peptide [17, 18], VirS is autophosphorylated. The phosphoryl group is then transferred to a conserved aspartate residue (D57) in VirR. Once phosphorylated, VirR directly stimulates or represses target gene expression by binding to two imperfect direct repeats, called VirR boxes, which are located upstream of the target genes [19, 20]. Genes that encode perfringolysin O (*pfoA*), α-clostripain (*ccp*) and three sRNAs (*vrr, virT,* and *virU*) are the only genes known to have upstream VirR boxes in *C. perfringens* strain 13 [14, 20, 21]. Of the sRNAs, the best studied is VR-RNA (encoded by *vrr*), which acts as a secondary regulatory molecule that controls the expression of 147 genes, including chromosomally encoded genes such as *plc* and *colA* (encoding κ-toxin), as well as the pCP13 encoded genes, *cpb2* (encoding β2-toxin) and *cnaB* (encoding a putative collagen adhesion) [22, 23]. By contrast, VirT regulates the expression of *pfoA* and *ccp* [14], while VirU controls the expression of *pfoA, ccp, vrr* and *virT* [21].

RevR has been shown to modulate virulence in a VirSR-independent manner since the expression of the VirSR-regulated virulence-associated genes, *plc* and *pfoA,* is unchanged in a *revR* mutant [15]. Sequence alignment and structure prediction of RevR has shown that it is similar to the response regulators PhoB from *Escherichia coli* and YycF from *Bacillus subtilis* [15]. Since a potential winged-helix-turn helix DNA binding domain was present within the C-terminal domain of RevR, it was proposed that RevR controls its regulon by binding to a DNA target [15]. Although the mechanism of action of RevR is still unclear, microarray analysis has revealed that RevR regulates many genes including those encoding hydrolytic enzymes (*nagL, nagH, nanI, nanJ* and *ccp*) and some sporulation genes. Importantly, a *revR* mutant was attenuated for virulence in the mouse myonecrosis model when compared to its wild-type parent [15].

In this study, to further characterize the VirSR and RevR regulatory systems, we carried out RNA-seq analysis of the *C. perfringens* strain JIR325, a derivative of strain 13, and its isogenic *virR* and *revR* regulatory mutants. Comparative strand-specific RNA-seq was used to identify all genes, sRNA and antisense transcripts that may be part of the VirSR or RevR regulons. These transcriptome analyses revealed that VirR is a global repressor and that RevR is a global activator. Importantly, VirR and RevR regulated several common genes, but in a reciprocal manner.

## Methods

### Bacterial strains and culture conditions

*C. perfringens* strain JIR325, a rifampicin and nalidixic acid resistant derivative of strain 13 [12], and its derivatives, JIR12364 (*virRΩTTermB*) and JIR12233 (*∆revRΩerm*) [15], were used in this study. The *virR* mutant was constructed by Targetron mutagenesis using the *virR* Targetron plasmid, pJIR3608, as outlined previously [24]. Potential mutants that had been cured of pJIR3608, but retained the Targetron insertion, were selected and analyzed by PCR and Southern blot hybridization as described previously [24]. One of these confirmed *virR* mutants, JIR12364, was selected for further analysis.

*C. perfringens* broth cultures were grown overnight at 37 °C in fluid thioglycolate (FTG) medium or in TPG broth medium (5 % (w/v) tryptone, 0.5 % (w/v) protease peptone and 0.1 % (w/v) sodium thioglycolate) with glucose added to a final concentration of 0.38 % (w/v) after sterilization. Agar cultures of *C. perfringens* were grown on nutrient agar (NA) (Oxoid or Difco, BD) supplemented with rifampicin (10 μg/ml), nalidixic acid (10 μg/ml) or erythromycin (50 μg/ml) under anaerobic conditions (10 % (v/v) H_2,_ 10 % (v/v) CO_2_ and 80 % (v/v) N_2_). Growth of these strains was determined by measuring the optical density (OD) at 600 nm using a WPA Biowave CO8000 cell density meter.

### Isolation of total RNA

*C. perfringens* strains were grown at 37 °C in TPG broth with an initial OD_600_ of 0.05 and cells were harvested by centrifugation at 8000 *g* for 10 min at room temperature, when the culture had reached late logarithmic phase (5 h; OD_600_ of 1.8–2.0). The harvested cells were resuspended and incubated in lysis buffer (10 mM Tris-HCI pH 7.5, 0.4 M sucrose, 5 mg/ml lysozyme) at 37 °C for 30 min. Following centrifugation at 16, 060 *g* for 10 min, the supernatant was discarded and total RNA was isolated from the cells using TRIzol® reagent (Invitrogen) as previously described [15]. To remove contaminating DNA, the RNA was treated with 4U of TURBO DNase (Ambion) at 37 °C for 90 min according to the manufacturer’s instructions. To test for DNA contamination, the RNA was used as a template for PCR without reverse transcription using oligonucleotide primers for the housekeeping gene, *rpoA* (Additional file 1: Table S1)*.* The PCR cycling conditions were as follows: 95 °C for 5 min, followed by 30 cycles of 95 °C for 1 min, 55 °C for 1 min, and 72 °C for 1 min. RNA quality was also validated by use of a Nanodrop spectrophotometer (Thermo Fisher) and only RNA with an absorbance at 260/280 nm ratio of > 1.9–2.1 was used for cDNA synthesis.

### PCR, reverse-transcription-PCR (RT-PCR) and quantitative real-time PCR (QRT-PCR)

All primers used in PCR, RT-PCR and QRT-PCR are listed in Additional file 1: Table S1. Standard PCR reactions were performed with Taq DNA polymerase (New England Biolabs). PCR cycling conditions were as shown earlier.

To examine gene expression and to validate RNA-seq data by QRT-PCR, 2 μg of total RNA was used for first strand cDNA synthesis using random hexamers (Promega) and AMV-reverse transcriptase (Promega). The negative control samples were prepared by replacing AMV-reverse transcriptase with nuclease-free water and were labeled as NRT (no reverse transcriptase). The reverse-transcription reaction was incubated for 1 h at 42 °C, followed by 10 min at 90 °C and 5 min on ice.

For QRT-PCR, each 25 μl reaction contained 12.5 μl of SYBR Green mastermix (Applied Biosystems), 1 μl of forward and reverse primers (Additional file 1: Table S1) at a final concentration of 5 μM, 1 μl of cDNA, and the appropriate amount of nuclease-free water. A standard curve for each gene was generated from genomic DNA of JIR325. PCR conditions were as follows: initial denaturation at 95 °C for 15 min, 40 cycles of 95 °C for 20 s, 50 °C for 30 s and 68° for 20 s. Reactions were confirmed as being the result of a single product by melting curve analysis. All QRT-PCRs were completed using the Mastercycler ep *Realplex* real time PCR system (Eppendorf) and were carried out on three independent biological replicates. The expression of each gene was normalized against the expression of the housekeeping gene, *rpoA,* from the same biological replicate, before comparative analysis.

### RNA-seq library preparation and RNA-seq analysis

Strand specific RNA-seq was carried out as before [25]. Briefly, for first strand synthesis (FSS), a 100 μl reaction that contained 8 μg of total RNA and 15 μg of random hexamers (Promega) was incubated at 65 °C for 5 min and placed on ice for 5 min. The reaction was then combined with 20 μl of 5X first strand buffer (Invitrogen), 10 μl of 100 mM of dithiothreitol (DTT) (Invitrogen), 5 μl of 10 mM of deoxynucleotide (dNTP) mix (Promega) and 2.5 μl of 40 U/μl RNasin (Promega) and incubated at 25 °C for 2 min. Subsequently, 5 μl of 200 U/μl SuperScript III was added to the reaction and incubated under the following conditions: 25 °C for 10 min, 42 °C for 50 min and 70 °C for 15 min. To remove excess dNTPs the FSS reaction was purified with a G-50 gel filtration spin column (GE Healthcare), according to the manufacturer’s instructions. Second strand synthesis was carried out as previously described [25], The resultant cDNA samples were used to construct cDNA libraries, where end-repair, A-tailing, ligation of adapter primers, size selection and pre-amplification were carried out with the Truseq RNA sample preparation kit version 1 (Illumina) as per the manufacturer’s instructions. The cDNA libraries were then sequenced as 100-mers using an Illumina Solexa GAIIx Sequencer by Micromon at Monash University. For each strain, two independent biological replicates were sequenced.

### RNA-seq data analysis

To analyze the RNA-seq data, the read pairs firstly were clipped of low quality regions and adaptor sequences, then aligned to the reference genome of *C. perfringens* strain 13 (GeneBank accession: NC_003366 and NC_003042) using SHRiMP alignment software (http://compbio.cs.toronto.edu/shrimp/). If a sequence read aligned to multiple locations on the genome, all alignments were kept for downstream statistical analysis and were referred to as a multi-hit read. The total number of aligned reads at each genomic position was counted using the Nesoni software package (http://www.vicbioinformatics.com/software.nesoni.shtml), a high-throughput sequencing data analysis toolset used to generate coverage depth plots, which can be visualized in the Artemis genome browser [26]. The mapped transcriptome data were then viewed, in a strand-specific manner, as a graph relative to the genome annotation.

Before analyzing the data, scatter plots of the expression levels of all genes measured by read counts were generated using Microsoft Excel to show the correlation and reproducibility between the biological replicates for each strain. Spearman's rho correlation coefficient between two replicates was then calculated using R code [27, 28]. Transcriptomic analysis was carried out using the Bioconductor edgeR analysis package [27], which uses a generalized linear model with a logarithmic link function and a negative binomial error distribution in the analysis. The dispersion parameter of the negative-binomial model was estimated per gene, and moderated with a global model of dispersion as a smoothly varying function of overall abundance (the "trend" option in edgeR). To minimize false positive results, a stringent cutoff of False Discovery Rate (FDR) <0.01 and log_2_ fold-change >1 was applied when identifying differentially expressed genes of the wild type compared to the mutant strains (Additional file 2: Table S2 & Additional file 3: Table S3).

With the Bioconductor “goseq” package [29], the differentially expressed gene lists (FDR <0.01; log_2_ fold-change > 1) of the *virR* and *revR* mutants were mapped to known pathways at Kyoto Encyclopedia of Genes and Genomes (KEGG) and Gene Ontology (GO) [29] so that over-represented categories in the gene lists could be identified. Finally, to determine genes that were regulated by both VirR and RevR, the differentially expressed gene lists were analyzed in the Vennt program (http://www.vicbioinformatics.com/software.vennt.shtml). Vennt generates dynamic Venn diagrams from differential expressed gene lists, which enables the identification of overlapping genes in the lists.

### RNA-seq data accession number

The gene expression data have been deposited in the NCBI Gene Expression Omnibus (GEO) database with the GEO series accession number GSE72137.

### Identification of the potential sRNAs

Many sRNA molecules are found within intergenic regions of bacterial genomes [30, 31]. Therefore, using the depth of coverage plot generated from the wild-type transcriptomic data, active transcription with a minimum length of 30 nucleotides and more than 10 reads within intergenic regions was recorded. A total of 93 previously unannotated transcripts were identified. These putative sRNAs were designated as SR1 to SR93 (Small RNA). These potential sRNAs included intergenic non-coding RNAs as well as 5’untranslated regions (UTRs), such as potential riboswitches, and 3’ UTRs.

### Northern blotting analysis

For Northern blot analysis, total RNA was isolated at hourly intervals (1 to 5 h) from wild-type cells cultured in TPG broth at 37 °C. Hybridization probes of the selected sRNAs were generated using the appropriate primers (Additional file 1: Table S1). A total of 10 μg of RNA from each time point was resolved by 1.5 % (w/v) agarose gel electrophoresis, transferred onto a nylon membrane and hybridized with specific probes. Northern blotting was carried out as previously described, with some modifications [32, 33]. DNA probes were generated by PCR using the genomic DNA of the wild type as a template and were labeled by use of the AlkPhos-direct kit (GE Healthcare Life Sciences). Primers used for probe generation are listed in Additional file 1: Table S1. Signals were detected using CDP-Star® chemiluminescence (Roche). The autoradiograms were developed using a BAS 2000 Bio-Imaging Analyzer (Fuji Photo Film Co., Ltd., Kanagawa, Japan). All Northern blot analyses were performed at least twice using independently derived RNA preparations to confirm the reproducibility of the results.

## Results

### VirR acts as a global negative regulator, whilst RevR acts as a global positive regulator

To further characterize the VirSR and RevR virulence-related regulatory systems, we carried out strand-specific RNA-seq analysis on the wild-type strain JIR325 and its respective *virR* and *revR* mutants, JIR12364 and JIR12233. RNA from each strain was extracted from cells cultured in TPG broth for 5 h, which corresponded to late logarithmic growth phase. Two independent biological replicates were sequenced for each strain. Approximately 90 % of the reads were retained after quality and adapter clipping, and of these reads, 97 % aligned to the strain 13 reference genome (wild type, ca. 9.3 M read-pairs per replicate; *virR* mutant, ca. 11.2 M read-pairs per replicate and *revR* mutant, ca. 12.7 M read-pairs per replicate). Note that because the rRNA was not depleted from the total RNA samples, many of these reads (92 %) were derived from the rRNA transcripts. Scatter plots were generated to demonstrate the reproducibility between the two biological replicates (Additional file 4: Figure S1A-C). With Spearman’s rho correlation coefficients of greater than 0.928, the data were deemed to be highly reproducible.

**Fig. 1:**
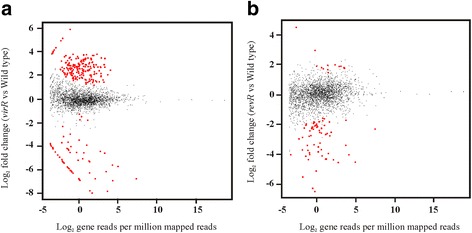
Relative gene expression of the *virR* and *revR* mutants compared to the wild type. Strand-specific RNA-seq libraries of the wild-type strain JIR325 and its *virR* and *revR* mutants were generated from RNA extracted from each strain. Comparative transcriptomic analysis was carried out and differentially expressed genes in the **a** *virR* and **b** *revR* mutants were identified using the “DESeq” R package. In the MA (log ratios versus mean average) plots that are shown, each point represents a gene. Red dots represent genes with a significant change in expression with a cutoff of FDR > 0.01 and an absolute log_2_ fold change > 1. Black dots denote genes with a fold change that is not within the range of the set cutoff. The “Y” axis represents the log_2_ fold change compared to the wild type and the “X” axis is the average log_2_ normalized counts in two independent biological replicates

In the *virR* mutant, 206 chromosomally encoded genes were found to be differentially expressed compared to the wild type (Additional file 2: Table S2). Of these genes, 95 % (195) were expressed at a higher level in the *virR* mutant compared to the wild type, with only 5 % (11) having lower expression in the *virR* mutant (Fig. 1a). These results indicate that VirR acts primarily as a global transcriptional repressor. Nevertheless, VirR positively regulated the expression of 11 chromosomally encoded genes (Additional file 2: Table S2), including five genes (*ccp, virT, virU, vrr* and *pfoA*) with upstream VirR boxes [20]. The six remaining genes, encoding transporter proteins (encoded by *pstC*, *fruA*, *nirC* and *cpe0947*), a phosphoribosymaninomidazole carboxylase catalytic subunit (*purE*) and a hypothetical protein (*cpe0844*) (Additional file 2: Table S2), did not contain upstream VirR boxes.

By contrast, in the *revR* mutant, 67 chromosomally encoded genes were found to be differentially expressed (Additional file 3: Table S3). Of these genes, 81 % (54) were expressed at a lower level in the *revR* mutant compared to the wild type, providing evidence that RevR acts primarily as a global transcriptional activator (Fig. 1b). The RevR regulon included genes encoding hyaluronidases (*nagH*, *nagI*, *nagJ*, *nagL*), a sialidase (*nanJ*), a protease (*cspB*) and a transcriptional repressor (*ctsR*) (Additional file 3: Table S3)*.* Furthermore, consistent with our previous microarray study [15], RevR enhanced the expression of genes encoding several hydrolytic enzymes including NagH, NagL and NanJ (Additional file 3: Table S3). The *revR* and *virR* genes were expressed at levels similar to wild type in the *virR* and *revR* mutants, respectively. In the *revR* mutant, the log_2_ fold change of *virR* was 0.425 with an FDR of 0.711, while in the *virR* mutant, the log2 fold change of *revR* was 0.0913 with an FDR or 0.9767. The FDRs of both genes were notably higher than the 0.01 threshold, indicating that they were not significantly different from the levels in the wild type. These results suggest that these genes are expressed independently.

**Fig. 2:**
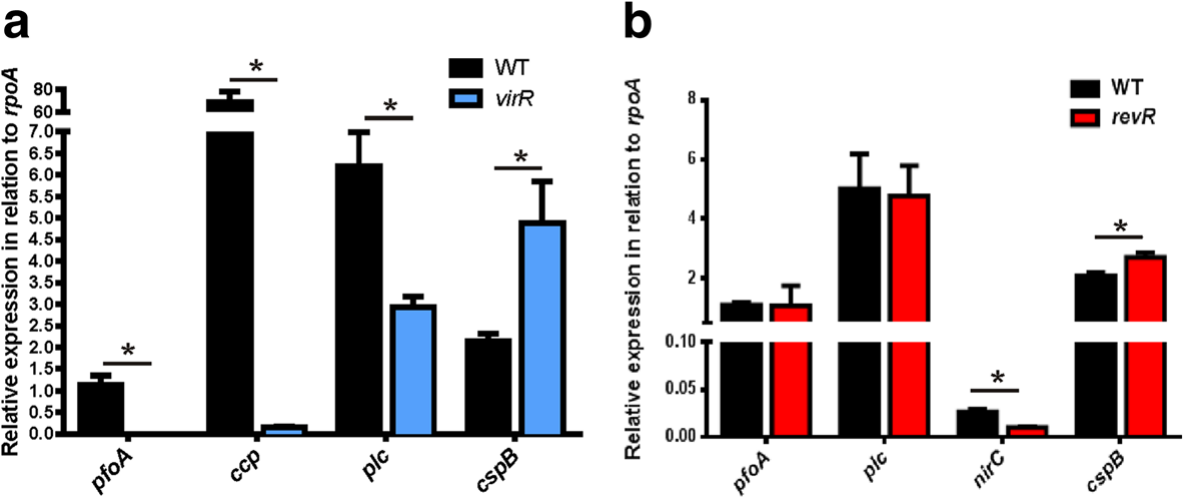
QRT-PCR validation of selected genes in the *virR* and *revR* mutants. RNA was extracted from cells grown in TPG broth for 5 h, which corresponded to the late logarithmic growth phase. RNA was converted to cDNA using reverse transcriptase, and was subsequently used as the template in QRT-PCR, which was performed to validate the relative expression of the *pfoA*, *ccp*, *plc*, and *cspB* genes in the *virR* mutant (**a**) and the expression of the *pfoA, plc, nirC* and *cspB* genes in the *revR* mutant (**b**). Expression levels are shown relative to the expression of the housekeeping gene, *rpoA,* and are the average of at least three independent biological replicates (*n* ≥ 3, mean ± SEM). The asterisk (*) denotes a statistically significant difference (*p ≤* 0.05) as calculated with the student’s unpaired *t*-test

QRT-PCR was employed to validate the expression of selected VirR-regulated genes (Fig. 2a). To encompass a range of expression changes, an up-regulated gene (*cspB*) and two down-regulated genes (*pfoA* and *ccp*) were selected for validation. In agreement with the RNA-seq data, QRT-PCR confirmed that the expression of the *pfoA* and *ccp* genes was reduced and that expression of *cspB* was increased in the *virR* mutant. The expression of the *plc* gene was also of interest, as it encodes the major toxin involved in gas gangrene [7]. With the stringent criteria used here (FDR < 0.01 and fold change > 2-fold), the expression of *plc* was not deemed to be changed. However, previous work has shown that α-toxin production is reduced in a *virR* mutant [12]. Closer inspection of the RNA-seq data showed that the *plc* gene did show weakly reduced expression (1.8-fold reduced expression; FDR = 0.04). Therefore, QRT-PCR was employed to further examine *plc* gene expression. The QRT-PCR confirmed that *plc* was down-regulated in the *virR* mutant compared to the wild type (Fig. 2a; 2.2-fold change, *P* < 0.05).

For validation of the *revR* RNA-seq expression data, QRT-PCR was performed on the *pfoA, plc, nirC* and *cspB* genes (Fig. 2b). These genes were selected as they represented genes whose expressions were either unchanged (*pfoA*, *plc*), down-regulated (*nirC*) or up-regulated (*cspB*) in the *revR* mutant. The QRT-PCR results (Fig. 2b) were in agreement with, and thereby validated, the RNA-seq data.

### Functional classification of differentially expressed genes

**Fig. 3:**
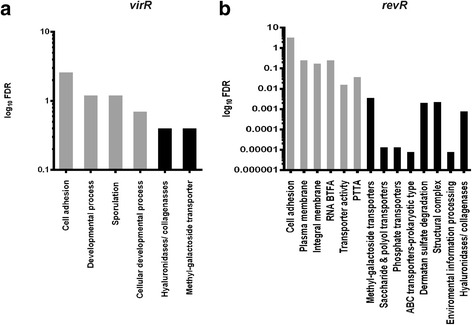
Gene Ontology (GO) and KEGG enrichment analysis. Bar charts denote the log_10_ FDR (False Discovery Rate) of over-represented GO (*grey*) and KEGG (*black*) categories in the **a** *virR* and **b** *revR* mutants as identified by the “Goseq” Bioconductor package. Abbreviations: RNA BTFA: RNA polymerase binding transcription factor activity; PTTA: phosphate transmembrane transporter activity

To allow functional analysis of the differentially expressed genes, they were classified into specific Gene Ontology (GO) groups [34] and pathways using the Kyoto Encyclopedia of Genes and Genomes (KEGG) [35]. These analyses indicated that many genes encoding components involved in sporulation and developmental processes were differentially expressed in the *virR* mutant (Fig. 3a), while genes encoding proteins associated with metabolite transport systems and membrane integrity were differentially expressed in the *revR* mutant (Fig. 3b).

Genes encoding proteins within the hyaluronidase/collagenase/cell adhesion functional category were significantly over-represented in the differentially expressed genes sets for both the *virR* and *revR* mutants (Fig. 3). In the *virR* mutant, the genes encoding NagH, NanJ, CPE0818 (Endo-beta-N-acetylglucosaminidase), CPE0866 (alpha N-acetylglucosaminidase), NagJ, NagL, a sialidase-like-protein (CPE1264), and a hypothetical protein (CPE1364) showed increased expression, whereas in the *revR* mutant genes encoding NagH, NanJ, NagJ, NagL, CPE0866, NagI and the hypothetical protein (CPE1876) showed reduced expression.

### Several genes are regulated by both VirR and RevR

**Fig. 4:**
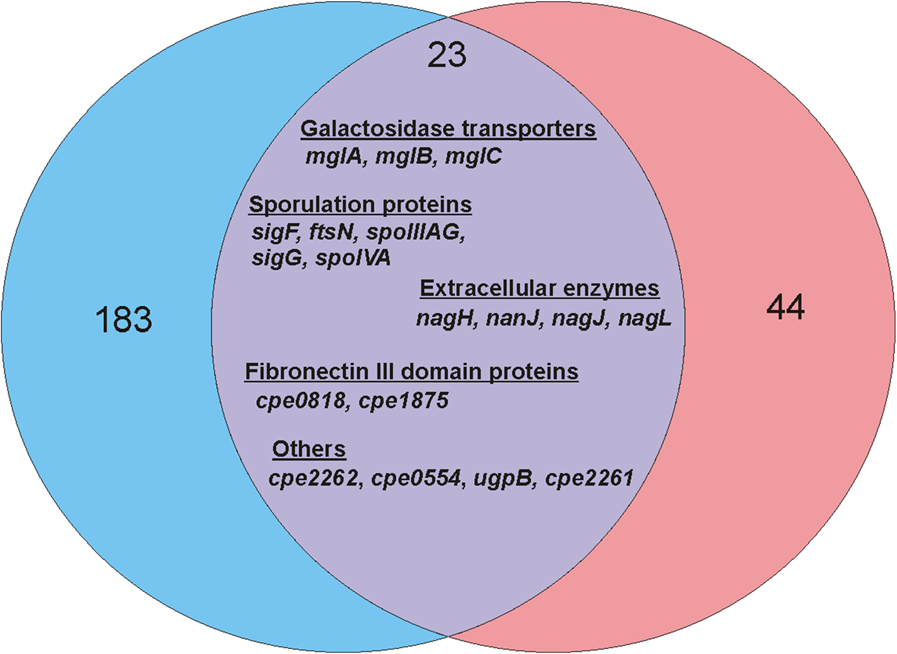
Genes that were regulated by both VirR and RevR. The Venn diagram generated with Vennt software represents the number of genes that were regulated by VirR (183; *blue*), RevR (44; *pink*) or both (23; *purple*). The 18 genes that were controlled by both regulators are indicated. The five genes that were regulated in a similar manner by VirR and RevR are not listed here

To reveal the distinctive roles of VirR and RevR, we identified genes that were regulated by only one or both of these response regulators. More genes were regulated solely by VirR (183) than by RevR (44) (Fig. 4). Genes encoding ferredoxin (CPE2511 and CPE1065), PTS system proteins (CPE2629, CPE2631 and CPE2632) and more than 30 sporulation-related proteins are examples of the genes that were regulated only by VirR (Additional file 2: Table S2). Genes encoding putative ABC transporters (CPE2031, CPE2081, CPE2082, and CPE0576-0578), a transcriptional repressor (CtsR), and a signal peptidase type I (CPE2295) were among the genes that were regulated only by RevR (Additional file 3: Table S3).

By contrast, 23 chromosomal genes were regulated by both VirR and RevR (Table 1). Eighteen of these genes were expressed at higher levels in the *virR* mutant and at lower levels in the *revR* mutant compared to the wild type (Table 1) (Fig. 4). Functional characterization of these 18 genes showed that they encoded proteins that were clustered into a few functional groups, namely proteins with fibronectin type III domains (CPE0818 and CPE1875), several extracellular hydrolytic enzymes (NagH, NanJ, NagJ and NagL), sporulation-associated proteins (SigF, FtsN, SpoIIIAG, SigG and SpoIVA), galactoside transporters (MglB, MglA and MglC) and three hypothetical proteins (Table 1). PSI-BLAST searches and InterProScan analysis of the hypothetical proteins showed that CPE2262 and CPE0554 contained a metalloprotease domain and that CPE2261 was a putative extracellular solute binding protein that may function in the initiation of sensory transduction pathways.

**Table 1:** Genes regulated by both VirR and RevR

Functional Group	Locus	Gene Name	Putative Product	Log_2_ fold change: *virR* vs WT^a^	Log_2_ fold change: *revR* vs WT^a^
Extracellular enzyme	*cpe0191*	*nagH*	hyaluronidase	2.07	−1.77
	*cpe0553*	*nanJ*	sialidase	2.15	−3.41
	*cpe1234*	*nagJ*	hyaluronidase	1.90	−2.10
	*cpe1523*	*nagL*	hyaluronidase	1.44	−2.07
Sporulation proteins	*cpe2048*	*sigF*	sporulation sigma factor	1.98	−1.98
	*cpe2146*	*ftsN*	Sporulation/cell division protein	1.56	−2.09
	*cpe1827*	*spoIIIAG*	sporulation protein AG	3.02	−4.03
	*cpe1761*	*sigG*	sporulation sigma factor	2.73	−2.06
	*cpe1753*	*spoIVA*	sporulation protein A	2.73	−2.44
Galactoside transporters	*cpe1341*	*mglB*	galactoside ABC transporter	2.42	−3.90
	*cpe1342*	*mglA*	galactoside ABC transporter	2.70	−3.71
	*cpe1343*	*mglC*	galactoside ABC transporter	2.69	−4.36
Fibronectin III domain proteins	*cpe1875*	*cpe1875*	fibronectin/fucosidase	1.40	−1.84
	*cpe0818*	*cpe0818*	endo-beta-N-acetylglucosaminidase	1.63	−2.26
Hypothetical proteins	*cpe1257*	*ugpB*	ABC transporter substrate binding protein	1.47	−3.51
	*cpe0554*	*cpe0554*	camelysin-like protein	2.45	−3.25
	*cpe2262*	*cpe2262*	has a camelysin M73 family peptidase domain	1.46	−2.20
	*cpe2261*	*cpe2261*	hypothetical protein	1.53	−1.93
Others^b^	*cpe2562*	*cspB*	protease	3.87	4.49
	*cpe0855*	*rubY*	rubrerythrin	1.42	1.66
	*cpe0584*	*fruA*	PTS fructose transporter	−1.48	−1.83
	*cpe0638*	*pstC*	phosphate ABC transporter permease	−1.27	−6.50
	*cpe0094*	*nirC*	nitrite transporter	−1.75	−5.13

^a^As defined by FDR <0.01; log_2_ fold change > 1

^b^Genes that are regulated by VirR and RevR in the same manner

Four genes were regulated by both VirR and RevR in the same manner: *cspB*, *rubY*, *fruA* and *pstC* (Table 1). The *cspB* and *rubY* genes were expressed at higher levels in both mutants, whereas the *fruA* and *pstC* genes were expressed at lower levels compared to the wild type (Table 1). These results suggested that VirR and RevR have both common and distinct functional roles in *C. perfringens.*

### VirR and RevR regulate genes located on the native plasmid, pCP13

Strains 13 and its derivatives harbor the 54.3 kb plasmid pCP13, which carries a β2-toxin gene, *cpb2* [36]. The VirSR regulatory system, *via* its secondary regulator, VR-RNA, is known to regulate the expression of two pCP13-encoded genes, *cpb2* and *cnaB* [37]. To further explore the role of VirR and RevR in the regulation of plasmid genes, the RNA-seq reads were also mapped to the 63 genes on pCP13.

**Fig. 5:**
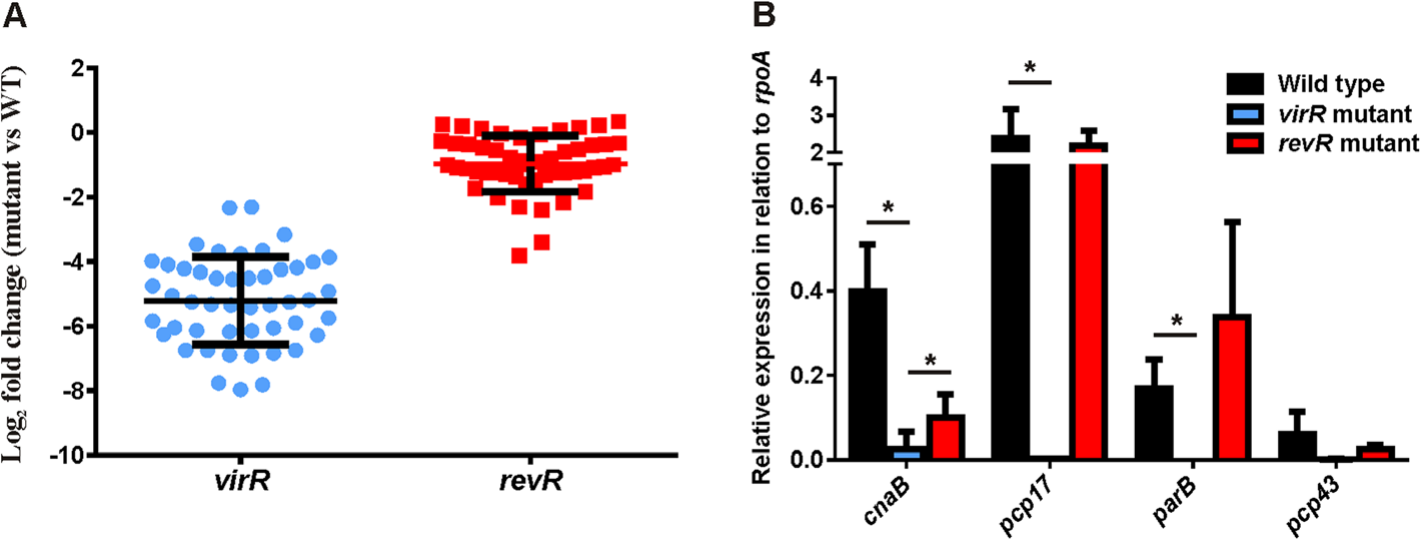
Relative expression of pCP13 genes in the *virR* and *revR* mutants compared to the wild type. **a** The expression levels of all plasmid-encoded genes in the *virR* (*blue*) and *revR* (*red*) mutants. Note that no FDR and fold-change cutoff criteria were applied to the data represented here. **b** Real-time PCR was performed to validate differentially expressed pCP13 plasmid genes in the *virR* and *revR* mutants. Expression levels were relative to the expression of the housekeeping gene, *rpoA,* and were the average of at least three independent biological replicates (*n* ≥ 3, mean ± SEM). The asterisk (*) denotes a statistically significant difference (*p ≤* 0.05) as calculated with the student’s unpaired *t*-test

The transcription of 47 of the 63 pCP13 genes was altered in the *virR* mutant (Additional file 5: Table S4). By contrast, only seven plasmid-encoded genes, including *cnaB,* were found to be positively regulated by RevR (FDR <0.01, log_2_ fold change > 1) (Additional file 6: Table S5). Analysis of the transcripts of all of the pCP13 genes by RNA-seq showed that the majority of the plasmid-encoded genes were expressed at lower levels in the *virR* and *revR* mutants, with the *virR* mutation having a greater effect on gene expression than the *revR* mutation (Fig. 5a). This observation was not due to pCP13 plasmid loss or instability, as the expression of a control gene, *pcp43*, was validated using QRT-PCR and showed statistically similar expression in the wild type and mutant strains (Fig. 5b). QRT-PCR validation was also performed on the *cnaB, pcp17* and *parB* transcripts*.* The results correlated with the RNA-seq data, which showed decreased expression of the *cnaB* gene in both mutants. The *pcp17* and *parB* genes*,* were down-regulated in the *virR* mutant, but their expression was not statistically different in the *revR* mutant (Fig. 5b).

### VirR and RevR regulate novel unannotated transcripts

**Fig. 6:**
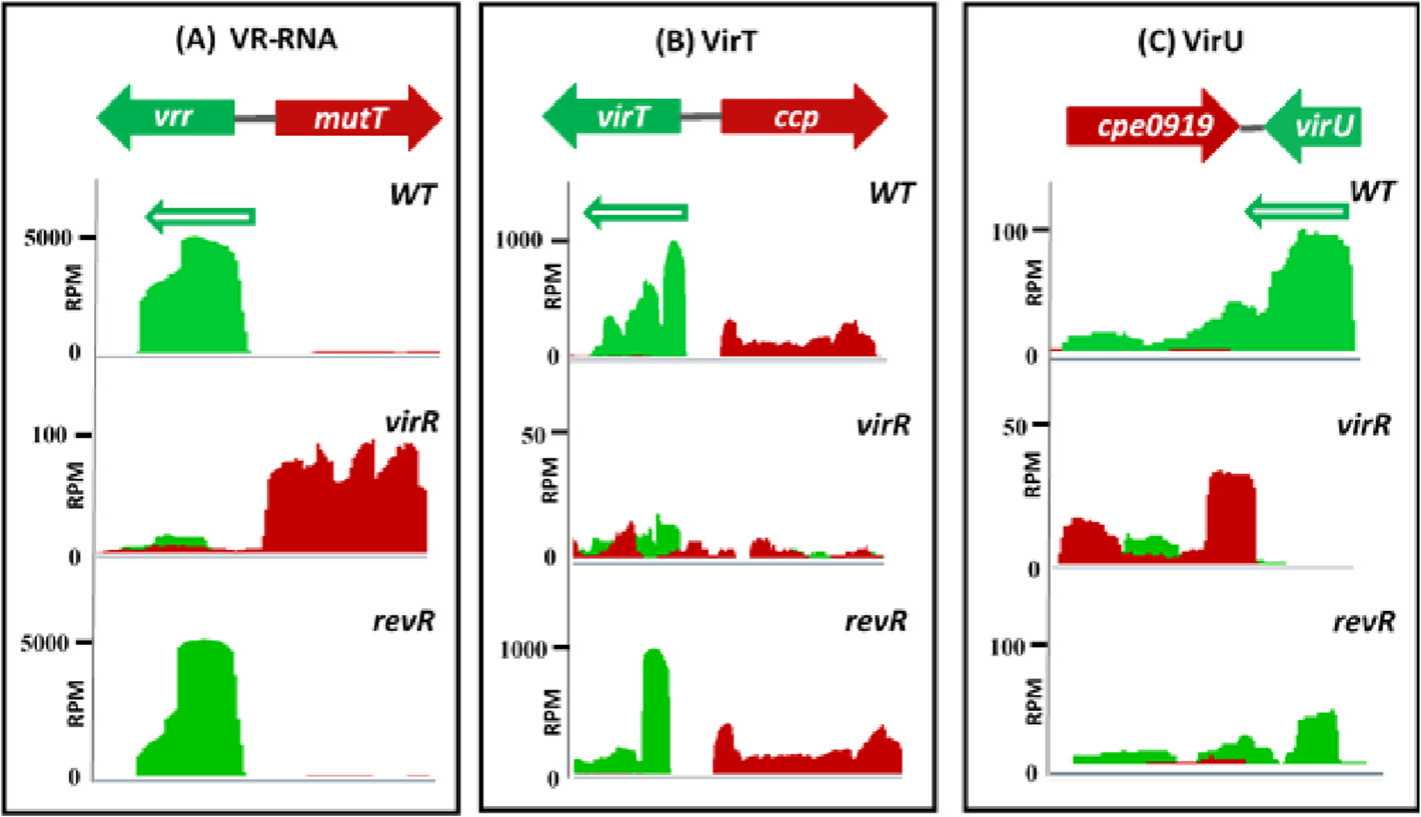
Expression levels and depth of coverage plots of VR-RNA, VirT and VirU. Depth of coverage plots of **a** VR-RNA (*cpe0957*), **b** VirT (*cpe0845*) and **c** VirU (*cpe0920*) in the wild-type strain, and the *virR* and *revR* mutants are shown as visualized in Artemis. The genetic organization of each sRNA is shown on the top of each figure. Mapped RNA-seq data are displayed as a plot showing sequence depth for the forward (*red*) and reverse (*green*) strand. In the depth of coverage plots, the Y axes represent the coverage depth in reads per million mapped reads (RPM). Expressed sRNAs are clearly seen as peaks in the depth of coverage plots

VirR regulates many genes by activating the transcription of sRNAs such as VR-RNA, VirT and VirU [13, 21]. To identify other potential sRNAs that may be regulated by VirR or RevR, the RNA-seq reads from each strain were mapped against the C. *perfringens* genome and analyzed by visual inspection in the Artemis genome viewer. A number of putative sRNAs were identified as strongly transcribed sections within intergenic regions. Importantly, the known VirR-regulated sRNAs, VR-RNA, VirT and VirU, had low or no detectable expression in the *virR* mutant compared to the wild type (Fig. 6), confirming previous studies [21, 38] and validating this approach.

**Fig. 7:**
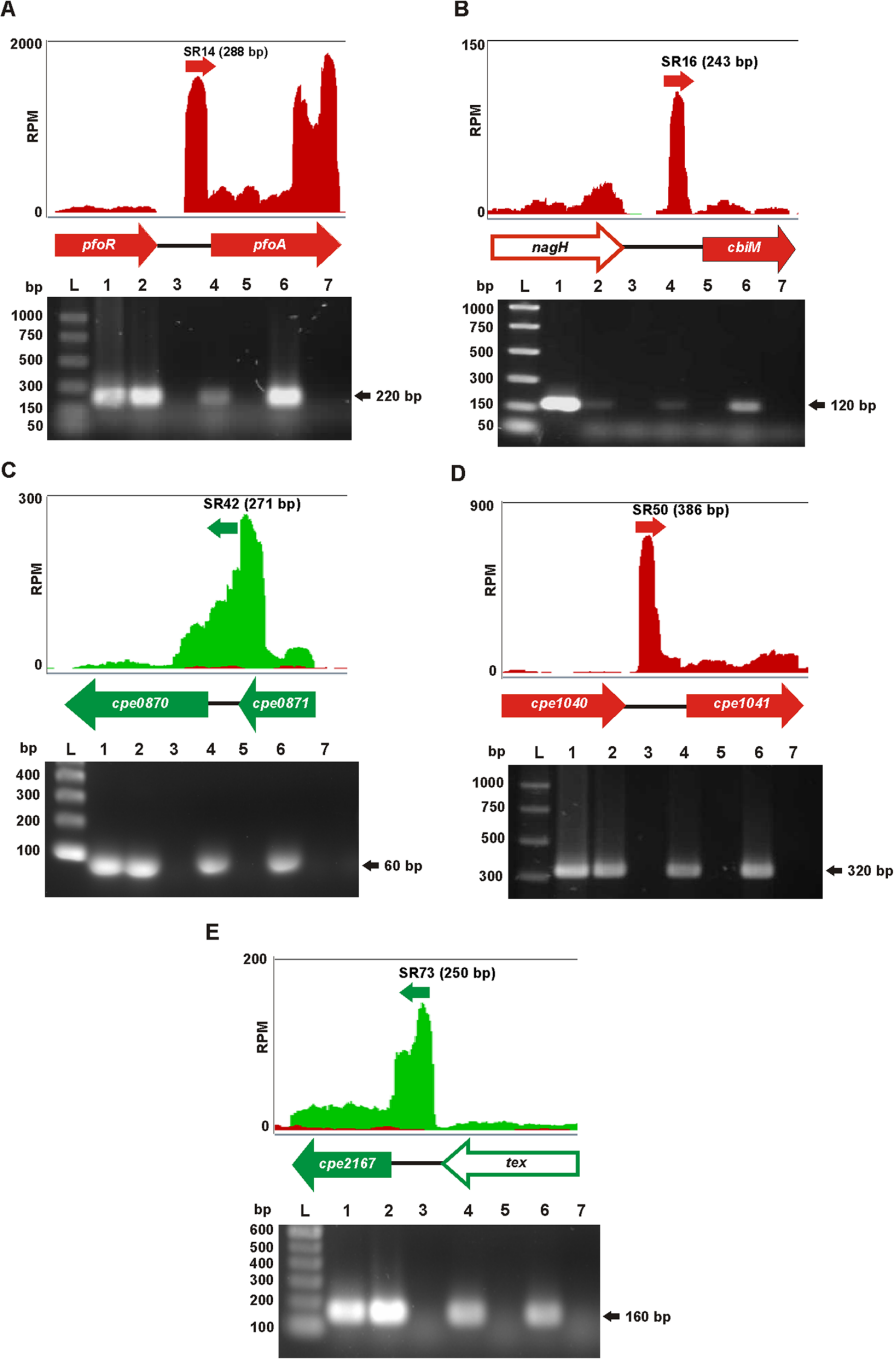
Validation of selected sRNA transcripts. Depth of coverage plots of **a** SR14, **b** SR16 **c** SR42, **d** SR50 and **e** SR73 together with their respective genetic organization and RT-PCR results are shown. Depth of coverage plots from the RNA-seq data of the wild-type JIR325 are depicted, where red and green graphs represent transcription on the sense- and antisense-strand, respectively. The Y axes show the coverage depth in reads per million mapped reads (RPM) and the X axes show the corresponding genetic organization. The approximate transcript lengths are indicated in brackets. Whole genes are represented by filled arrows, while open arrows indicate partial genes. RT-PCR was carried out to validate these unannotated transcripts. L, PCR marker ladder; 1, DNA control; 2, wild-type RNA with RT reaction; 3, wild-type RNA with no RT; 4, *virR* mutant RNA with RT; 5, *virR* mutant RNA with no RT; 6, *revR* mutant RNA with RT; 7, *revR* mutant RNA with no RT. The estimated sizes of the RT-PCR products (in kb) are shown on the left of the agarose gel images

**Fig. 8:**
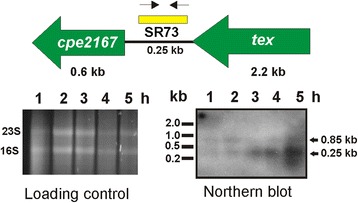
Time-course Northern blotting analysis of SR73. RNA was isolated from the wild-type JIR325 cultured in TPG broth at the indicated times (1, 2, 3, 4 and 5 h). A total of 10 μg of RNA extracted from the wild-type strain was used in Northern blotting analysis to examine the expression of SR73. The loading control shows the total RNA samples that were separated by agarose gel electrophoresis and later transferred to a Nylon membrane for Northern blot analysis. The genes flanking SR73 are indicated as green arrows, while the yellow bar shows the position of the SR73 probe. The probe was generated using forward (→) and reverse (←) primers as designated in the gene map. The hybridization of the probe to the target RNA was detected by CDP-*Star* chemiluminescence. The black solid arrow marks the transcript that hybridized to the probe and the estimated size of the transcripts (in kb) is shown on the right of the Northern blot image

Detailed analysis of the genome–wide transcript data identified 93 transcripts that were longer than 30 nucleotides and located within intergenic regions. They were designated SR1 to SR93 (Additional file 7: Table S6). To confirm expression of these putative sRNAs, four transcripts were selected for validation by RT-PCR (Fig. 7) and one by Northern blot analysis (Fig. 8). These sRNAs were chosen based on their location near putative virulence-related genes, as sRNAs often regulate the expression of adjacent genes [39].

RT-PCR was carried out using RNA purified from 5 h cultures of the wild-type strain JIR325, the *virR* and *revR* mutant strains to replicate the conditions of the RNA-seq analysis. The signals of the selected sRNAs on the wild-type depth of coverage plot were used to estimate the size of each sRNA. RT-dependent transcripts with the predicted sizes (Fig. 7a-e) were detected using specific primer pairs spanning SR14*,* SR16*,* SR42, SR50 and SR73 in both wild-type and mutant strains. The absence of products in the no RT reactions confirmed that the products were not derived from DNA contamination.

One of the four sRNAs, SR73, was also analyzed by Northern blotting (Fig. 8). Two transcripts, 0.85 kb and 0.25 kb in size, hybridized with the SR73-specific probe (Fig. 8). The faint 0.85 kb transcript was only observed in the RNA samples isolated during early growth phase (1 and 2 h), indicating that SR73 (0.25 kb) was co-transcribed with the downstream gene, *cpe2167* (0.60 kb) during this growth phase*.* The 0.25 kb band (SR73) was observed in increasing intensity in the RNA preparations from mid- and late-logarithmic phases (3, 4 and 5 h). By contrast, the 0.85 kb band (SR73 and *cpe2167*) was detected only during the early logarithmic phase (1 and 2 h). Bioinformatic analysis of the SR73 coding sequence indicated that SR73 was a putative Flavin mononucleotide (FMN) riboswitch. Its location upstream of *cpe2167*, in conjunction with the Northern blot results implied that SR73 could potentially repress the expression of *cpe2167* through the formation of a premature terminator during mid- to late- logarithmic growth phase.

The intergenic transcripts (SR1 to SR93) that were potentially regulated by VirR and RevR were also identified by performing comparative analysis of the RNA-seq data. In the *virR* mutant, only two sRNAs, SR45 and SR67, were up-regulated compared to the wild type, while in the *revR* mutant, four sRNAs, SR7, SR27, SR77 and SR82, were significantly down-regulated (Table 2 & Additional file 3: Table S3). These results indicated that in the wild-type strain the expression of SR45 and SR67 was usually repressed by VirR and that the expression of SR7, SR27, SR77 and SR82 was normally activated by RevR.

**Table 2:** Differentially expressed sRNAs in the *virR* and *revR* mutants

Strain	sRNA	Gene 1	Product 1^a,b^	Gene 2	Product 2^a,b^	log_2_ fold change
*virR* mutant	SR45	*eutQ*	entanolamine utilization protein	*btuE*	glutathione peroxidase	2.19
	SR67	*valS*	valine-tRNA ligase	*cpe1920*	hypothetical protein	1.85
*revR* mutant	SR7	*mviM*	dehydrogenase	*nirC*	nitrite transporter	−3.74
	SR27	*cpe0454*	membrane protein	*cpe0455*	alkaline phosphatase-like protein	−2.54
	SR77	*ddpA*	oligopeptide transporter	*narK*	nitrate extrusion protein	−2.62
	SR82	*bglB*	beta-glucosidase	*putA*	aldehyde dehydrogenase	−1.97

^a^As defined by FDR < 0.01; log_2_ fold change >1

^b^Gene 1 and gene 2 are used to designate the genes on each side on the postulated sRNA gene

## Discussion

In this study, we have used strand-specific RNA-seq to investigate the transcription of genes in a gas gangrene-causing isolate of *C. perfringens*. In particular, we analyzed gene expression in the *virR* and *revR* regulatory mutants compared to the wild type. Through this analysis, we have identified a subset of genes that are regulated by both response regulators, but in a reciprocal manner. VirR was found to repress the expression of these genes, while RevR enhanced their expression. The proteins encoded by these genes can be classified into the extracellular enzymes, sporulation proteins and galactoside transporters functional groups. Both VirR and RevR may be independently repressing and enhancing the expression of these genes, respectively. Alternatively, the VirR and RevR regulatory systems may converge and confer opposite effects on one of their common secondary regulators, thereby leading to opposite outcomes on the expression of the target genes. The reciprocal regulation of genes has been described in *Pseudomonas aeruginosa* [40, 41], where multiple regulatory systems exert opposite effects on the expression of the sRNA RsmZ and therefore cause different downstream consequences to the RsmZ target genes [40].

Previous studies suggested that the VirR and RevR regulatory cascades were stimulated by different external signals. The signal that was proposed to activate the VirSR system is the AgrD-derived quorum sensing autoinducer peptide (AIP) [17, 18], while the RevR regulatory system was hypothesized to be triggered by inorganic phosphate levels [15]. Hence, reciprocal regulation of similar genes by VirR and RevR is unlikely to be the result of these systems responding differently to the same signal. Moreover, it is unlikely that the VirR and RevR pathways converge on the known sRNA molecules (VR-RNA, VirU and VirT) that are directly regulated by the VirSR regulatory system, since the RNA-seq results showed that the *revR* mutation had no effect on the expression of these sRNAs. Therefore, we postulate that VirR and RevR independently regulate these genes in a reciprocal manner.

The RNA-seq results provided evidence that VirR is a global negative regulator that represses the expression of 195 genes and enhances the expression of only 11 genes. It is not known how VirSR negatively regulates the expression of these genes, as no VirR boxes [20] were found in their promoter regions. Therefore, it is likely that negative regulation involves a secondary intermediate like VR-RNA, since it has previously been shown that the VirSR system regulates as many as 147 genes through this sRNA molecule [22]. Of the 11 genes that were positively regulated by VirR, five genes (*pfoA, vrr, virU, virT* and *ccp*) were preceded by VirR boxes and thus were controlled directly by the binding of VirR to these sites [19, 20]. This result is consistent with a previous microarray study [22]. The positive regulation of the *cpe0094* gene by VirR was indirect and may be attributed to the VR-RNA molecule, as previously described [22]. How VirR positively regulates the remaining five genes (*cpe0844, purE, cpe0947, fruA* and *pstC*) is still unclear.

In a previous study, several virulence-associated genes were found to be under the positive control of the VirSR-VR-RNA cascade during the early- and mid-logarithmic growth phase. The expression of genes including *plc, colA, nagL, nanI, nanJ, nanE* and *nanA* was affected by the *vrr* (VR-RNA) mutation [22]. By contrast, our RNA-seq results showed that the expression of *plc, colA, nanI*, *nanE* and *nanA* was unchanged in the *virR* mutant, whereas *nagL* and *nanJ* were repressed by VirR at late-logarithmic growth phase. These contrasting results may have arisen because the *vrr* mutation has a more direct effect on the expression of its target genes compared to the mutation of *virR*, which encodes the positive regulator of *vrr.* An alternative explanation is that the RNA samples used in both transcriptomic studies were prepared from different media and growth phases. In addition, different analysis methods were employed in the two studies [22].

The transcriptomic results revealed that as many as 36 sporulation-related genes were negatively regulated by VirR (Additional file 2: Table S2). By contrast, four sporulation-associated genes were positively controlled by RevR (Additional file 3: Table S3). Similarly, previous microarray analysis indicated that expression of sporulation-related genes was affected by RevR [42]. Sporulation is a critical event in *C. perfringens-*mediated food poisoning. Resistant spores enhance the survival of the sporulating *C. perfringens* strains in the small intestine [43] and the enterotoxin (CPE) that is produced during sporulation has been shown to cause the symptoms of acute food poisoning [44, 45]. However, *C. perfringens* strain 13 is a CPE-negative gas gangrene strain that has a low sporulation efficiency [46]. It has been proposed that the absence of the spore coat proteins CotJB and CotJC, or the presence of a premature stop codon in the master sporulation regulatory gene *spo0A,* is responsible for the sporulation defect in this strain [36]. We have shown that the expression of sporulation genes was affected by the *virR* mutation, including genes encoding spore maturation proteins (SpoA/B), stage II to V proteins and sporulation sigma factors (SigF, SigG and SigK). The *revR* mutation also affected the expression of some sporulation-related genes, albeit to a lesser extent. Of these genes, *sigF* and *sigG* were significantly downregulated in the *revR* mutant, suggesting that these genes are normally activated by RevR. Note that the *virR* mutation has the opposite effect on these genes. These two sigma factors have been shown to be essential for sporulation, with SigF being critical for CPE production [43]. A VirR-independent sRNA, VirX, has also been reported to negatively regulate the expression of sporulation sigma factor genes, including *sigE, sigF* and *sigK,* and mutation of *virX* leads to an increase in sporulation and CPE production in the food-poisoning strain SM101 [47]. The RNA-seq data showed that there was no significant difference in the expression of *virX* (*cpe0646*) in the *virR* mutant compared to the wild type. Hence, VirSR may work together with VirX to negatively regulate sporulation-related genes in sporulating *C. perfringens* strains.

The RNA-seq data showed that RevR behaves primarily as a positive regulator. The mechanism by which RevR regulates its target genes is as yet unknown. However, considering that it shares a high level of similarity with PhoB, a response regulator in the phosphate (P_i_) signal transduction system, we previously postulated that RevR may function in a phosphate-dependent manner [15]. In *E. coli*, it has been suggested that the two-component proteins PhoR and PhoB sense phosphate levels by monitoring the activity of the phosphate-specific ATP-binding cassette transporter PstSCAB [48]. The RNA-seq results showed that genes (*cpe0636 – cpe0641*), which are located directly upstream of *revR*, and encode putative PstSCAB transporter proteins, were positively regulated by RevR.

These data indicate that RevR controls at least 44 genes independently of VirR. Consistent with previous findings, *nagH, nanJ, nagL* and *cpe1753* (stage IV sporulation protein A) were expressed at a lower level in the *revR* mutant [15]. Other genes, including *nanI, ccp* and *cpe0185,* which were previously found to be expressed at a higher level in the *revR* mutant [15], showed no significant change in their expression levels in the current study. The expression of other genes also differed from that reported in a previous study [15]. The discrepancy may be due to different transcriptomic approaches, the use of different media and the fact that the RNA was isolated at different time points.

KEGG and GO analysis of the differentially expressed genes in both mutants revealed the potential physiological roles of the VirSR and RevR regulatory systems. However, further experiments need to be conducted to substantiate the proposed functions of these candidate genes and any potential roles in virulence. Half of the GO over-represented categories in the *virR* mutant were involved in cell developmental processes that lead to changes in the cell over time, indicating that the VirSR regulatory system not only has a role in virulence, but also influences the growth of *C. perfringens.* In the *revR* mutant, other than the phosphate transmembrane transporter system, cell membrane proteins and transporters were significantly over-represented in the differentially regulated genes, suggesting that RevR may have a major role in maintaining membrane integrity and controlling the uptake and/or export of nutrient/ions in *C. perfringens.*

VirSR is one of the most important regulatory systems in *C. perfringens* and this regulon has been characterized using different approaches [21, 22, 49, 50]. However, none of these studies addressed the transcripts within intergenic regions or on pCP13, except for one study, which provided preliminary evidence that the VirSR system affects the expression of the *cnaB* and *cpb2* genes on pCP13 [37]. Microarray analysis failed to detect transcription of other pCP13 genes [22], probably due to the low expression levels of transcripts from these regions and the narrow dynamic range of the microarray technique. In this study, comparative RNA-seq analysis showed that both VirR and RevR alter the transcription of pCP13 genes, with VirR having a more noticeable effect. The expression of 47 pCP13 genes was at least four times lower in the *virR* mutant compared to the wild-type strain. It is possible that some of these changes may be due to indirect effects of the response regulator(s) on plasmid copy number. Previously, the *cnaB* and *cpb2* genes were found to be negatively and positively regulated, respectively, by the VirSR system in a VR-RNA dependent manner during the mid to late logarithmic growth phases [37]. However, the results obtained in this study indicated that both genes were positively regulated by the VirSR regulatory system during late logarithmic growth phase and have provided evidence that the VirR and RevR regulatory systems control not only chromosomally-encoded genes, but also the expression of plasmid-encoded genes*.*

It is now well established that sRNAs regulate diverse processes in bacteria, ranging from stress adaptation to the control of central and secondary metabolism and virulence processes [31, 51]. By employing strand-specific RNA-seq, we have identified 93 novel transcripts encoded on the wild-type chromosome (Additional file 7: Table S6). Of these previously unannotated transcripts, 30 corresponded to previous *in silico*-predicted sRNAs of *C. perfringens* strain 13 [52] (Additional file 7: Table S6), strongly supporting their approach. Comparative analysis of the expression of these sRNAs in the wild type and the mutants revealed that VirSR and RevR regulated the expression of two and four sRNAs, respectively (Table 2). Note that although RNA-seq depth of coverage plots can provide a preview of the transcription pattern, which helps in the prediction of putative sRNAs, Northern blots need to be conducted to confirm whether these putative sRNAs are independent molecules or read-through transcripts from close adjacent genes. Our studies have laid the foundation for further analysis of the sRNAs of *C. perfringens*. The primary remaining challenge is to decipher the functional roles of these putative sRNAs. These studies will identify new regulatory systems, and will likely extend many established signaling pathways. For example, a potential sRNA, SR14, was identified upstream of the *pfoA* gene (Fig. 7a). Sequence analysis of the potential coding region of SR14 showed that this putative sRNA was located downstream of the VirR binding sites, the VirR boxes, and the *pfoA* promoter. Although there is no experimental evidence to show that SR14 affects the expression of *pfoA,* the potential involvement of a sRNA would suggest that the regulation of PFO production may be much more complex than previously thought. Irrespective of the outcome of such studies, the identification of these novel transcripts has greatly refined the annotation of the *C. perfringens* genome, aiding to the current knowledge of this important pathogenic bacterium.

## Conclusions

In summary, in this study RNA-seq was used to analyze the transcriptional activity of *C. perfringens* strain JIR325 and its isogenic *virR* and *revR* mutants. The VirR and RevR regulons include chromosomally encoded genes, pCP13-encoded genes and previously unidentified sRNAs. VirR was found to behave primarily as a global negative regulator, while RevR acted as a global positive regulator. Furthermore, our data indicate that a small subset of genes are regulated by both the VirSR and RevR pathways. Further investigation is needed to answer many questions arising from this study, such as the relative importance of the VirR and RevR pathways and the functional roles of the novel sRNA transcripts. Nevertheless, the results reported in this study have significantly enhanced our understanding of these virulence-related regulatory systems.

## Additional files

Additional file 1: Table S1. Oligonucleotide primers used in this study. (DOCX 14 kb) Additional file 2: Table S2. Genes with significant change in expression between the wild type and *virR* mutant as identified by the edgeR analysis package. (DOCX 29 kb) Additional file 3: Table S3. Genes with significant change in expression between the wild type and *revR* mutant as identified by the edgeR analysis package. (DOCX 18 kb) Additional file 4: Figure S1. Validation of replicate RNA-seq data (TIF 242 kb) Additional file 5: Table S4. Plasmid pCP13 genes that are differentially expressed in the *virR* mutant compared to the wild type. (DOCX 15 kb) Additional file 6: Table S5. Plasmid pCP13 genes that are differentially expressed in the *revR* mutant compared to the wild type. (DOCX 13 kb) Additional file 7: Table S6. Small RNAs identified from the wild-type depth of coverage plot. (DOCX 17 kb)
