# Supplementary material for: RNA-seq analysis of virR and revR mutants of Clostridium perfringens
Source: BMC Genomics. 2016 May 23;17:391. doi: 10.1186/s12864-016-2706-2 (PMC4877802; doi:10.1186/s12864-016-2706-2)
Supplement: Additional file 1: Table S1. — Oligonucleotide primers used in this study. (DOCX 14 kb) [file 12864_2016_2706_MOESM1_ESM.docx]

**Table S1:** Oligonucleotide primers used in this study.

| Primer | Sequence (5’ to 3’) | Description |
| --- | --- | --- |
| JRP2479 | CCATCTGTTTTTATATCTGCTCCAGTA | *rpoA,* forward primer |
| JRP2480 | GGAAGGTGAAGGACCAAAAACTATT | *rpoA,* reverse primer |
| JRP3385 | GGAGCAATTGATGAGTTAGTGTCTAAGT | *pfoA,* forward primer |
| JRP3386 | TTCTGAATATTGAGTTCTTGCTGGTAA | *pfoA,* reverse primer |
| JRP4418 | CAAGCAATACCACATTGGGCT | *ccp,* forward primer |
| JRP4419 | GTTTTCCATAACCACTTCTTAATCTATAAGC | *ccp,* reverse primer |
| JRP5406 | TGTAAGGCGCTTATTTGTGC | *plc,* forward primer |
| JRP5407 | AAGCGTAGACTTTAGTTGATGCC | *plc,* reverse primer |
| JRP5929 | TCATGTTGCAGGAATTGCAT | *cspB,* forward primer |
| JRP5930 | AGGCCGCCCTTAAACTATTC | *cspB,* reverse primer |
| JRP5931 | TGCATTTGTTATCGCTGGTT | *nirC,* forward primer |
| JRP5932 | CCCCCTCCTACCATATTTCC | *nirC,* reverse primer |
| JRP5933 | GGGGGAAGGTTCTTCCAATA | *cna,* forward primer |
| JRP5934 | CCAGCATCAACTTGCATTTC | *cna,* reverse primer |
| JRP5935 | TCACAATCAATTGGGGGAGT | *cpb2,* forward primer |
| JRP5936 | TCATCTCCCCATAACCAATGA | *cpb2,* reverse primer |
| JRP5937 | GCTAATGCACAATCAAGAGAGC | *parB,* forward primer |
| JRP5938 | TGGAACTTTTTCTCCATTTTCC | *parB,* reverse primer |
| JRP5998 | CAGATTCAAATTTGTCTGTAGAGCTT | *pcp43,* forward primer |
| JRP5999 | AAAAGTTAATGAAACTGCACTCCA | *pcp43,* reverse primer |
| JRP5617 | TATCGAGGATCCTGCCATTTGGCTCTTATGAA | SR50*,* forward primer |
| JRP5618 | ACGCTGGAGCTCGGTTATGGTGATAGATATAACTAC | SR50*,* reverse primer |
| JRP5218 | TTTGTACTTTAAGCCACTG | SR16*,* forward primer |
| JRP5219 | TAGAGGTTATAAAAATTCTAAAAAA | SR16*,* reverse primer |
| JRP5210 | ATTTTGGTTCCTCTGGTT | SR42*,* forward primer |
| JRP5211 | ATCTTGAGGGGATGAGTT | SR42*,* reverse primer |
| JRP5224 | TTAGAGAGAGTTTAGGGA | SR14, forward primer |
| JRP5225 | CCTCATTTATTAAGCTCAT | SR14*,* reverse primer |
| JRP5455 | TTCCGAGGCCGACAGTATAG | SR73, forward primer |
| JRP5456 | ATTTTGTCCCCTCCCAAAAG | SR73, reverse primer |
