# Supplementary material for: RNA-seq analysis of virR and revR mutants of Clostridium perfringens
Source: BMC Genomics. 2016 May 23;17:391. doi: 10.1186/s12864-016-2706-2 (PMC4877802; doi:10.1186/s12864-016-2706-2)
Supplement: Additional file 2: Table S2. — Genes with significant change in expression between the wild type and virR mutant as identified by the edgeR analysis package. (DOCX 29 kb) [file 12864_2016_2706_MOESM2_ESM.docx]

**Table S2:** Genes with significant change in expression between the wild type and *virR* mutant as identified by the edgeR analysis package.

| Locus Tag | Log_2_ Fold change^a^ | FDR | Gene | Product |
| --- | --- | --- | --- | --- |
| CPE0034 | 2.35 | 9.39E-06 |  | hypothetical protein |
| CPE0037 | 2.8 | 4.31E-06 | *cobW* | CobW/P47K family protein |
| CPE0038 | 2.91 | 3.73E-07 |  | hypothetical protein |
| CPE0039 | 2.85 | 1.68E-05 |  | hypothetical protein |
| CPE0050 | 1.74 | 8.75E-04 |  | hypothetical protein |
| CPE0067 | 3.46 | 6.12E-12 |  | hypothetical protein |
| CPE0068 | 1.64 | 1.43E-03 | *glgC* | glucose-1-phosphate adenylyltransferase |
| CPE0069 | 1.89 | 1.70E-04 | *glgD* | glycogen biosynthesis protein |
| CPE0094 | -1.75 | 1.86E-03 | *nirC* | nitrite transporter NirC |
| CPE0105 | 2.52 | 4.70E-05 |  | hypothetical protein |
| CPE0130 | 2.22 | 1.83E-04 |  | hypothetical protein |
| CPE0140 | 1.41 | 2.40E-03 |  | DNA helicase IV |
| CPE0141 | 5.16 | 1.75E-05 |  | hypothetical protein |
| CPE0163 | -5.71 | 2.26E-23 | *pfoA* | perfringolysin O |
| CPE0191 | 2.07 | 3.26E-06 | *nagH* | hyaluronidase |
| CPE0203 | 2.11 | 9.42E-04 |  | hypothetical protein |
| CPE0204 | 2.74 | 7.74E-07 |  | acetyltransferase |
| CPE0206 | 2.03 | 1.89E-04 |  | hypothetical protein |
| CPE0209 | 2.36 | 4.22E-07 |  | hypothetical protein |
| CPE0237 | 2.49 | 3.65E-08 | *napA* | Na+/H+ antiporter |
| CPE0240 | 2.17 | 1.63E-04 |  | hypothetical protein |
| CPE0241 | 2.04 | 1.54E-04 |  | flagellar motor protein MotB |
| CPE0246 | 2.5 | 3.18E-05 |  | dnaJ domain protein |
| CPE0247 | 2.6 | 3.75E-04 | *grpE* | heat shock protein GrpE |
| CPE0248 | 2.91 | 4.98E-08 | *dnaK* | molecular chaperone DnaK |
| CPE0249 | 2.81 | 8.31E-08 |  | hypothetical protein |
| CPE0252 | 2.44 | 2.84E-08 |  | hypothetical protein |
| CPE0253 | 2.05 | 1.10E-06 |  | hypothetical protein |
| CPE0254 | 1.77 | 2.92E-04 |  | hypothetical protein |
| CPE0264 | 2.98 | 3.28E-07 |  | hypothetical protein |
| CPE0266 | 1.79 | 3.14E-05 |  | beta-hexosamidase A |
| CPE0287 | 3.25 | 6.81E-11 |  | phage-related single-strand DNA binding protein |
| CPE0331 | 2.78 | 1.93E-09 |  | hypothetical protein |
| CPE0363 | 1.71 | 4.83E-04 |  | hypothetical protein |
| CPE0383 | 2.21 | 5.29E-06 |  | hypothetical protein |
| CPE0388 | 1.29 | 4.01E-03 | *deoB* | phosphopentomutase |
| CPE0430 | 1.51 | 5.61E-04 | *sipS* | type I signal peptidase |
| CPE0511 | 2.26 | 2.51E-03 |  | hypothetical protein |
| CPE0515 | 4.17 | 2.72E-03 |  | hypothetical protein |
| CPE0543 | 1.78 | 6.19E-04 |  | permease |
| CPE0553 | 2.15 | 2.09E-04 | *nanJ* | exo-alpha-sialidase |
| CPE0554 | 2.45 | 2.03E-06 |  | hypothetical protein |
| CPE0556 | 3.23 | 3.53E-04 |  | NAD(P)H dehydrogenase |
| CPE0564 | 2.03 | 8.16E-06 | *spoVD* | stage V sporulation protein D |
| CPE0584 | -1.48 | 1.08E-03 | *fruA* | PTS fructose transporter |
| CPE0587 | 2.38 | 4.03E-03 |  | hypothetical protein |
| CPE0638 | -1.27 | 8.87E-03 | *pstC* | phosphate ABC transporter permease |
| CPE0647 | 2.2 | 4.23E-03 |  | hypothetical protein |
| CPE0651 | 2.76 | 7.85E-09 |  | hypothetical protein |
| CPE0681 | -2.97 | 4.23E-03 | *purE* | phosphoribosylaminoimidazole carboxylase, catalytic subunit |
| CPE0818 | 1.63 | 5.36E-03 |  | endo-beta-N-acetylglucosaminidase |
| CPE0844 | -3.9 | 1.43E-14 |  | hypothetical protein |
| CPE0845 | -4.58 | 3.24E-19 | *virT* | small regulatory RNA |
| CPE0846 | -4.43 | 2.01E-18 | *ccp* | alpha-clostripain |
| CPE0855 | 1.42 | 2.77E-03 | *rubY* | rubrerythrin |
| CPE0866 | 2.11 | 1.72E-05 |  | alpha-N-acetylglucosaminidase |
| CPE0867 | 2.71 | 1.08E-03 |  | hypothetical protein |
| CPE0886 | 3.5 | 9.81E-13 |  | LysM domain protein |
| CPE0919 | 3.27 | 1.42E-05 |  | hypothetical protein |
| CPE0920 | -5.11 | 2.34E-05 | *virU* | small regulatory RNA |
| CPE0947 | -1.78 | 3.79E-04 |  | transmembrane symporter |
| CPE0952 | 2.63 | 5.86E-07 |  | hypothetical protein |
| CPE0957 | -5.59 | 1.34E-26 | *vrr* | small regulatory RNA |
| CPE1023 | 2.77 | 3.18E-08 |  | hypothetical protein |
| CPE1046 | 2.57 | 3.15E-06 |  | putative alpha-glucosidase |
| CPE1047 | 2.59 | 1.30E-04 |  | hypothetical protein |
| CPE1062 | 2.54 | 3.92E-03 |  | electron transport complex protein RnfG |
| CPE1063 | 4.36 | 7.76E-04 | *nqrD* | electron transport complex RsxE subunit |
| CPE1065 | 2.39 | 2.91E-03 |  | ferredoxin |
| CPE1078 | 2.44 | 1.68E-03 |  | hypothetical protein |
| CPE1087 | 3.62 | 4.53E-11 |  | hypothetical protein |
| CPE1164 | 2.84 | 2.00E-05 |  | hypothetical protein |
| CPE1230 | 2.92 | 2.33E-05 |  | hypothetical protein |
| CPE1234 | 1.9 | 1.17E-05 | *nagJ* | hyaluronidase |
| CPE1235 | 2.43 | 3.26E-08 |  | hypothetical protein |
| CPE1242 | 2.01 | 6.93E-06 | *cspL* | cold shock protein |
| CPE1254 | 1.86 | 2.80E-04 | *gltB* | oxidoreductase |
| CPE1255 | 2.1 | 2.02E-03 | *hydG* | ferredoxin-NADP reductase |
| CPE1256 | 3.11 | 1.38E-10 | *bdhB* | NADPH-dependent butanol dehydrogenase |
| CPE1257 | 1.47 | 5.00E-03 |  | hypothetical protein |
| CPE1264 | 2.45 | 5.25E-08 |  | sialidase-like protein |
| CPE1266 | 2.18 | 1.10E-06 |  | beta-galactosidase |
| CPE1293 | 2.21 | 4.06E-03 |  | tetratricopeptide repeat protein |
| CPE1330 | 2.76 | 3.01E-08 |  | hypothetical protein |
| CPE1331 | 3.46 | 4.94E-09 | *rubY* | rubrerythrin |
| CPE1332 | 2.6 | 2.69E-06 | *spoVR* | stage V sporulation protein R |
| CPE1333 | 2.87 | 2.47E-05 |  | hypothetical protein |
| CPE1334 | 3.45 | 9.49E-10 | *prkA* | serine protein kinase |
| CPE1341 | 2.42 | 3.34E-08 | *mglB* | galactoside ABC transporter |
| CPE1342 | 2.7 | 5.80E-08 | *mglA* | galactose/methyl galaxtoside transporter ATP-binding protein |
| CPE1343 | 2.69 | 2.63E-09 | *mglC* | beta-methylgalactoside transporter inner membrane component |
| CPE1353 | 1.7 | 6.42E-04 |  | hypothetical protein |
| CPE1356 | 3.93 | 6.21E-03 |  | hypothetical protein |
| CPE1364 | 2.32 | 2.98E-07 |  | hypothetical protein |
| CPE1380 | 1.42 | 2.10E-03 |  | hypothetical protein |
| CPE1381 | 2.78 | 3.15E-07 |  | hypothetical protein |
| CPE1384 | 2.05 | 9.04E-04 |  | hypothetical protein |
| CPE1404 | 2.68 | 1.43E-07 |  | hypothetical protein |
| CPE1409 | 3.09 | 3.36E-06 |  | electron transport complex protein RnfC |
| CPE1410 | 2.84 | 1.71E-06 |  | Na-translocating NADH-quinone reductase Nqr2 subunit |
| CPE1423 | 2.08 | 5.36E-05 | *sas2* | small acid-soluble spore protein C2 |
| CPE1455 | 3.88 | 9.50E-03 |  | hypothetical protein |
| CPE1523 | 1.44 | 2.73E-03 | *nagL* | hyaluronidase |
| CPE1530 | 3.04 | 7.81E-09 |  | hypothetical protein |
| CPE1553 | 3.55 | 2.04E-07 | *sipS* | signal peptidase I |
| CPE1558 | 2.88 | 2.66E-05 |  | di-trans, poly-cis-decaprenylcistransferase |
| CPE1559 | 2.25 | 2.61E-04 |  | diapophytoene dehydrogenase |
| CPE1647 | 2.04 | 1.63E-04 |  | hypothetical protein |
| CPE1648 | 3.37 | 2.93E-11 | *spoVB* | stage V sporulation protein B |
| CPE1649 | 2.05 | 1.97E-03 |  | hypothetical protein |
| CPE1652 | 1.59 | 6.20E-03 |  | hypothetical protein |
| CPE1657 | 2.62 | 4.15E-06 | *comEC* | late competence protein |
| CPE1663 | 2.04 | 1.13E-06 |  | hypothetical protein |
| CPE1665 | 2.79 | 1.22E-09 |  | PhoH family protein |
| CPE1667 | 2.25 | 8.66E-07 |  | single-strand binding protein |
| CPE1704 | 3.48 | 8.04E-07 | *comM* | Mg chelatase-like protein |
| CPE1727 | 1.66 | 3.74E-04 |  | hypothetical protein |
| CPE1753 | 2.93 | 1.29E-09 | *spoIVA* | stage IV sporulation protein A |
| CPE1760 | 2.68 | 3.90E-06 |  | hypothetical protein |
| CPE1761 | 2.73 | 1.51E-08 | *sigG* | sporulation sigma factor SigG |
| CPE1768 | 3.3 | 4.51E-10 | *sigK* | sporulation sigma factor SigK |
| CPE1769 | 2.65 | 6.10E-05 | *spoVD* | stage V sporulation protein D |
| CPE1782 | 2.08 | 3.25E-03 |  | PRC-barrel domain protein |
| CPE1801 | 4.05 | 4.87E-03 | *dacB* | D-alanyl-D-alanine carboxypeptidase |
| CPE1802 | 1.95 | 1.37E-04 |  | hypothetical protein |
| CPE1803 | 2.8 | 2.81E-09 |  | hypothetical protein |
| CPE1808 | 3.82 | 5.22E-14 |  | tyrosine recombinase XerD |
| CPE1809 | 2.93 | 9.45E-07 | *spoIIM* | stage II sporulation protein M |
| CPE1826 | 2.77 | 6.28E-07 | *spoIIIAH* | stage III sporulation protein AH |
| CPE1827 | 3.02 | 5.66E-07 | *spoIIIAG* | stage III sporulation protein AG |
| CPE1829 | 3.81 | 4.98E-08 | *spoIIIAE* | stage III sporulation protein AE |
| CPE1830 | 3.35 | 3.66E-06 | *spoIIIAD* | stage III sporulation protein AD |
| CPE1831 | 4.96 | 4.37E-05 | *spoIIIAC* | stage III sporulation protein AC |
| CPE1832 | 5.92 | 3.51E-08 | *spoIIIAB* | stage III sporulation protein SpoAB |
| CPE1833 | 2.49 | 3.98E-06 | *spoIIIAA* | stage III sporulation protein AA |
| CPE1874 | 3.71 | 1.05E-12 | *ctc* | 50S ribosomal protein L25 |
| CPE1875 | 1.4 | 9.50E-03 |  | hypothetical protein |
| CPE1900 | 3.7 | 3.46E-05 |  | hypothetical protein |
| CPE1903 | 1.89 | 6.97E-03 |  | small acid-soluble spore protein beta |
| CPE1920 | 3 | 1.37E-10 |  | tetratricopeptide repeat protein |
| CPE1949 | 2.63 | 7.27E-08 |  | hypothetical protein |
| CPE1961 | 1.74 | 2.35E-03 |  | sporulation protein YunB |
| CPE1985 | 2.2 | 3.71E-06 |  | TspO/MBR family protein |
| CPE2010 | 2.59 | 1.47E-07 | *cotS* | spore coat protein |
| CPE2021 | 2.49 | 2.31E-03 |  | sporulation protein YqfC |
| CPE2037 | 2.92 | 1.29E-10 |  | 50S ribosomal protein L25/general stress protein Ct |
| CPE2039 | 4.37 | 5.35E-04 |  | hypothetical protein |
| CPE2040 | 2.47 | 4.05E-08 | *spoIIP* | stage II sporulation protein P |
| CPE2048 | 1.98 | 3.03E-06 | *sigF* | sporulation sigma factor SigF |
| CPE2049 | 1.62 | 3.40E-04 | *spoIIAB* | anti-sigma F factor |
| CPE2050 | 1.56 | 3.59E-04 | *spoIIAA* | anti-sigma F factor antagonist |
| CPE2051 | 2.42 | 1.88E-07 |  | ATP-dependent protease |
| CPE2054 | 3.78 | 2.48E-12 |  | cation-transporting ATPase |
| CPE2064 | 2.65 | 1.05E-04 |  | small acid-soluble spore protein C1 |
| CPE2134 | 2.3 | 2.33E-04 |  | peptidase, M50 family |
| CPE2135 | 2.37 | 1.72E-07 |  | hypothetical protein |
| CPE2146 | 1.56 | 3.49E-04 | *ftsN* | sporulation/cell division protein |
| CPE2169 | 2.84 | 2.98E-07 |  | peptidase |
| CPE2178 | 2.59 | 7.23E-04 |  | hypothetical protein |
| CPE2179 | 3.16 | 8.44E-10 |  | sporulation protein YyaC |
| CPE2181 | 3.08 | 5.44E-05 | *spoIIID* | stage III sporulation protein D |
| CPE2182 | 3.45 | 1.27E-13 |  | putative peptidase |
| CPE2183 | 2.29 | 1.17E-05 | *spoIID* | stage II sporulation protein D |
| CPE2213 | 1.78 | 5.35E-04 | *cphA* | cyanophycin synthetase |
| CPE2214 | 3.59 | 1.61E-05 | *cphB* | cyanophycinase |
| CPE2215 | 2.72 | 3.45E-09 |  | lysM domain protein |
| CPE2218 | 2.75 | 1.28E-08 | *cotS* | spore coat protein CotS |
| CPE2224 | 2.59 | 3.72E-05 |  | hypothetical protein |
| CPE2225 | 2 | 1.62E-04 |  | hypothetical protein |
| CPE2226 | 3.38 | 1.95E-09 |  | hypothetical protein |
| CPE2227 | 2.24 | 2.22E-04 | *apbA* | thiamin biosynthesis lipoprotein |
| CPE2255 | 3 | 3.70E-10 |  | hypothetical protein |
| CPE2261 | 1.52 | 2.76E-03 |  | hypothetical protein |
| CPE2262 | 1.67 | 1.45E-03 |  | hypothetical protein |
| CPE2263 | 1.46 | 8.93E-03 |  | hypothetical protein |
| CPE2288 | 3.1 | 1.38E-07 |  | prepilin peptidase dependent protein A |
| CPE2352 | 2.46 | 5.74E-07 |  | hypothetical protein |
| CPE2353 | 3.36 | 3.31E-10 |  | spore cortex-lytic enzyme |
| CPE2445 | 3.45 | 2.99E-09 |  | metal-dependent hydrolase |
| CPE2454 | 1.89 | 4.43E-05 | *csfB* | csfB protein |
| CPE2455 | 3.6 | 1.56E-05 |  | amidohydrolase |
| CPE2473 | 1.68 | 1.28E-04 | *spoIIE* | stage II sprulation protein E |
| CPE2476 | 2.86 | 1.90E-04 |  | hypothetical protein |
| CPE2477 | 3.23 | 8.59E-10 |  | sporulation protein YabP |
| CPE2482 | 3.12 | 1.05E-04 |  | stage V sprulation protein T |
| CPE2504 | 3.19 | 3.30E-09 |  | hypothetical protein |
| CPE2508 | 2.54 | 2.11E-07 |  | hypothetical protein |
| CPE2511 | 3.16 | 3.23E-07 | *fer* | ferredoxin |
| CPE2515 | 2.91 | 5.37E-10 |  | hypothetical protein |
| CPE2516 | 2.78 | 1.10E-08 |  | hypothetical protein |
| CPE2519 | 1.2 | 9.50E-03 |  | hypothetical protein |
| CPE2532 | 3 | 3.72E-05 | *spmB* | spore maturation protein B |
| CPE2533 | 2.57 | 1.81E-04 | *spmA* | spore maturation protein A |
| CPE2547 | 2.68 | 3.43E-04 |  | hypothetical protein |
| CPE2559 | 3.36 | 1.30E-09 |  | hypothetical protein |
| CPE2562 | 3.87 | 8.57E-03 | *cspB* | protease CspB |
| CPE2575 | 2.71 | 1.46E-06 |  | [Fe] hydrogenase |
| CPE2576 | 2.38 | 1.52E-05 |  | 3-methyladenine DNA glycosylase |
| CPE2586 | 3.73 | 2.95E-09 |  | hypothetical protein |
| CPE2596 | 1.81 | 1.61E-04 |  | hypothetical protein |
| CPE2628 | 3.48 | 2.54E-09 |  | preprotein translocase subunit YajC |
| CPE2629 | 3.78 | 6.26E-10 |  | PTS system protein |
| CPE2630 | 3.71 | 1.98E-12 |  | mannose permease IIm |
| CPE2631 | 3.49 | 2.82E-12 |  | PTS system protein |
| CPE2632 | 2.92 | 1.17E-08 |  | PTS system protein |
| CPE2649 | 2.93 | 2.35E-08 |  | sporulation protein YyaC |
| SR45 | 2.19 | 5.36E-03 |  | Region between CPE0910 and CPE0911 |
| SR67 | 1.85 | 8.15E-03 |  | Region between CPE1919 and CPE1920 |

^a^ Fold-change is calculated as the *virR* mutant expression level over the wild-type expression level as defined by FDR <0.01 and log_2_ fold change >1. Positive and negative values represent gene expression up-regulated and down-regulated in the *virR* mutant compared to the wild type, respectively.
