# Supplementary material for: RNA-seq analysis of virR and revR mutants of Clostridium perfringens
Source: BMC Genomics. 2016 May 23;17:391. doi: 10.1186/s12864-016-2706-2 (PMC4877802; doi:10.1186/s12864-016-2706-2)
Supplement: Additional file 3: Table S3. — Genes with significant change in expression between the wild type and revR mutant as identified by the edgeR analysis package. (DOCX 18 kb) [file 12864_2016_2706_MOESM3_ESM.docx]

**Table S3:** Genes with significant change in expression between the wild type and *revR* mutant as identified by the edgeR analysis package.

| Locus Tag | Log_2_ Fold change^a^ | FDR | Gene | Product |
| --- | --- | --- | --- | --- |
| CPE0085 | -2.87 | 1.92E-03 | *mdh* | alcohol dehydrogenase |
| CPE0090 | -4.02 | 6.22E-06 |  | dehydrogenase |
| CPE0091 | -3.7 | 4.52E-06 | *iolE* | myo-inositol catabolism protein |
| CPE0092 | -4.14 | 2.20E-05 |  | symporter YidK |
| CPE0093 | -5.52 | 9.27E-05 |  | myo-inositol 2-dehydrogenase |
| CPE0094 | -5.13 | 1.01E-10 | *nirC* | nitrite transporter NirC |
| CPE0096 | -2.98 | 9.58E-03 |  | propionate CoA-transferase |
| CPE0097 | -2.8 | 4.70E-04 | *acdS* | acyl-CoA dehydrogenase |
| CPE0113 | -4.53 | 5.02E-11 |  | hypothetical protein |
| CPE0114 | -4.04 | 5.30E-11 |  | hypothetical protein |
| CPE0191 | -1.77 | 1.04E-03 | *nagH* | hyaluronidase |
| CPE0251 | -1.63 | 5.95E-03 |  | hypothetical protein |
| CPE0455 | -2.68 | 6.11E-04 |  | alkaline phosphatase-like protein |
| CPE0507 | -2.32 | 1.26E-03 |  | RNA polymerase sigma factor |
| CPE0553 | -3.41 | 8.11E-05 | *nanJ* | exo-alpha-sialidase |
| CPE0554 | -3.25 | 1.28E-06 |  | hypothetical protein |
| CPE0573 | -4.37 | 7.60E-10 |  | hypothetical protein |
| CPE0576 | -4.56 | 5.02E-11 |  | ABC transporter |
| CPE0577 | -3.95 | 3.13E-10 |  | ABC transporter permease |
| CPE0578 | -4.32 | 5.54E-10 |  | ABC transporter |
| CPE0584 | -1.83 | 3.95E-03 |  | PTS system |
| CPE0636 | -2.59 | 3.13E-06 | *pstS1* | phosphate ABC transporter phosphate-binding protein |
| CPE0637 | -6.28 | 5.54E-10 | *pstS2* | phosphate ABC transporter phosphate-binding protein |
| CPE0638 | -6.5 | 9.00E-11 | *pstC* | phosphate ABC transporter permease |
| CPE0639 | -3.37 | 7.12E-06 | *pstA* | phosphate ABC transporter permease |
| CPE0640 | -3.84 | 9.81E-09 | *pstB* | phosphate transporter ATP-binding protein |
| CPE0641 | -4.32 | 1.22E-11 | *phoU* | phosphate transporter PhoU |
| CPE0642 | -2.75 | 3.55E-05 | *revR* | DNA-binding response regulator |
| CPE0693 | -4.11 | 3.13E-10 |  | hypothetical protein |
| CPE0734 | 1.75 | 3.86E-03 | *naoX* | pyridine nucleotide-disulphide oxidoreductase |
| CPE0735 | 1.88 | 3.55E-03 |  | putative inner membrane protein |
| CPE0793 | 2.95 | 1.26E-03 |  | iron(III) dicitrate ABC transporter |
| CPE0818 | -2.26 | 4.46E-04 |  | endo-beta-N-acetylglucosaminidase |
| CPE0854 | 1.98 | 4.71E-04 | *aspC* | aminotransferase class V |
| CPE0855 | 1.66 | 8.13E-03 | *rubY* | rubrerythrin |
| CPE0881 | -2.11 | 8.41E-03 | *nagI* | hyaluronidase |
| CPE1098 | 1.72 | 4.55E-03 |  | hypothetical protein |
| CPE1099 | 1.88 | 9.47E-03 |  | hypothetical protein |
| CPE1173 | 1.78 | 5.11E-03 |  | hypothetical protein |
| CPE1234 | -2.1 | 1.53E-03 | *nagJ* | hyaluronidase |
| CPE1257 | -3.51 | 1.35E-07 |  | hypothetical protein |
| CPE1341 | -3.9 | 3.93E-07 | *mglB* | galactoside ABC transporter |
| CPE1342 | -3.71 | 3.59E-05 | *mglA* | galactose/methyl galaxtoside transporter ATP-binding protein |
| CPE1343 | -4.36 | 2.55E-07 | *mglC* | beta-methylgalactoside transporter inner membrane component |
| CPE1502 | -1.87 | 3.23E-03 |  | hypothetical protein |
| CPE1523 | -2.07 | 8.91E-04 | *nagL* | hyaluronidase |
| CPE1534 | -2.77 | 3.55E-05 |  | PTS system, sucrose-specific IIBC component |
| CPE1535 | 1.95 | 3.94E-03 |  | hypothetical protein |
| CPE1621 | -2.27 | 3.06E-03 |  | hypothetical protein |
| CPE1753 | -2.44 | 2.32E-04 | *spoIVA* | stage IV sporulation protein A |
| CPE1761 | -2.06 | 6.38E-03 | *sigG* | sporulation sigma factor SigG |
| CPE1827 | -4.03 | 8.41E-03 | *spoIIIAG* | stage III sporulation protein AG |
| CPE1875 | -1.84 | 9.92E-03 |  | hypothetical protein |
| CPE1876 | -2.28 | 1.11E-03 |  | hypothetical protein |
| CPE2031 | -2.25 | 2.77E-03 |  | ABC transporter |
| CPE2048 | -1.98 | 1.11E-03 | *sigF* | sporulation sigma factor SigF |
| CPE2081 | -2.19 | 8.12E-04 |  | ABC transporter |
| CPE2082 | -2.19 | 3.96E-03 |  | ABC transporter |
| CPE2146 | -2.09 | 4.70E-04 | *ftsN* | sporulation/cell division protein |
| CPE2261 | -1.93 | 2.77E-03 |  | hypothetical protein |
| CPE2263 | -2.2 | 8.12E-04 |  | hypothetical protein |
| CPE2295 | -2.53 | 6.83E-04 | *lepW* | signal peptidase type I |
| CPE2303 | 1.87 | 8.13E-03 |  | heavy metal-transporting ATPase |
| CPE2318 | 1.46 | 8.01E-03 |  | pyridoxamine kinase |
| CPE2444 | 1.79 | 3.08E-03 | *ctsR* | transcriptional repressor |
| CPE2554 | -1.59 | 7.77E-03 | *glpF* | glycerol uptake facilitator protein |
| CPE2562 | 4.49 | 3.19E-03 | *cspB* | protease CspB |
| SR7 | -3.74 | 5.99E-06 |  | Region between CPE0093 and CPE0094 |
| SR27 | -2.54 | 9.96E-04 |  | Region between CPE0454 and CPE0455 |
| SR77 | -2.62 | 2.50E-06 |  | Region between CPE2273 and CPE2274 |
| SR82 | -1.97 | 5.14E-03 |  | Region between CPE2309 and CPE2310 |

^a^ Fold-change is calculated as the *revR* mutant expression level over the wild-type expression level as defined by FDR <0.01 and log_2_ fold change >1. Positive and negative values represent gene expression up-regulated and down-regulated in the *revR* mutant compared to the wild type, respectively.
