# Supplementary figures and images for: RNA-seq analysis of virR and revR mutants of Clostridium perfringens
Source: BMC Genomics. 2016 May 23;17:391. doi: 10.1186/s12864-016-2706-2 (PMC4877802; doi:10.1186/s12864-016-2706-2)

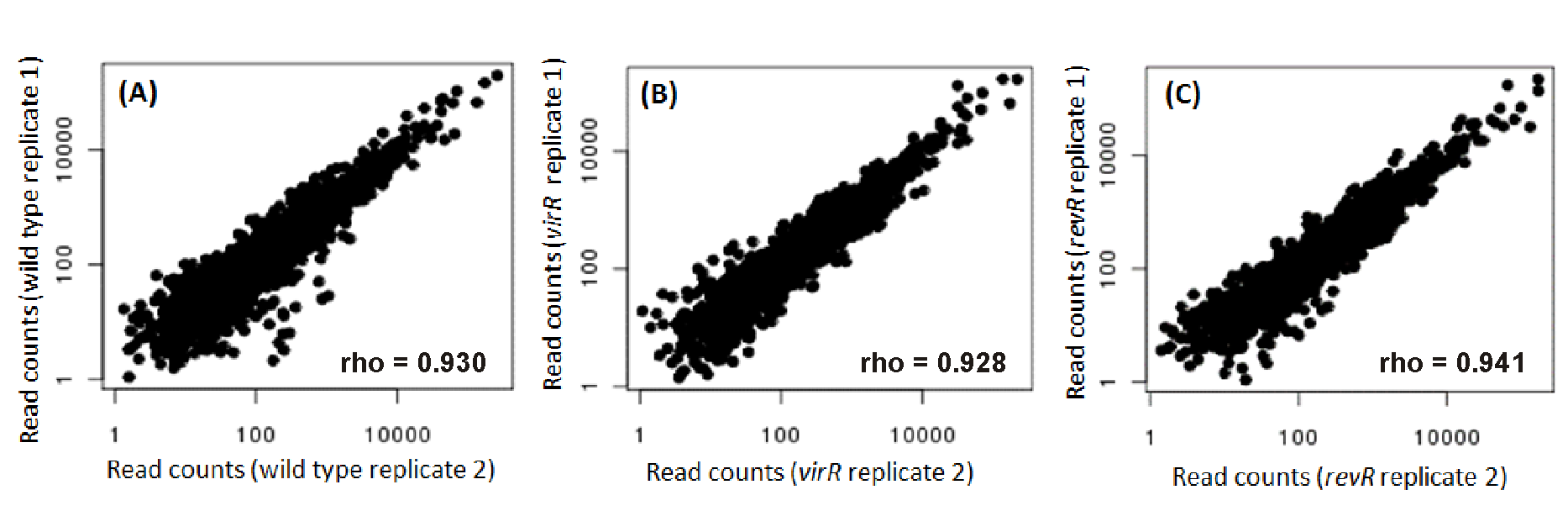

Supplement: Additional file 4: Figure S1. — Validation of replicate RNA-seq data (TIF 242 kb) [file 12864_2016_2706_MOESM4_ESM.tif]
