# Supplementary material for: RNA-seq analysis of virR and revR mutants of Clostridium perfringens
Source: BMC Genomics. 2016 May 23;17:391. doi: 10.1186/s12864-016-2706-2 (PMC4877802; doi:10.1186/s12864-016-2706-2)
Supplement: Additional file 5: Table S4. — Plasmid pCP13 genes that are differentially expressed in the virR mutant compared to the wild type. (DOCX 15 kb) [file 12864_2016_2706_MOESM5_ESM.docx]

**Table S4:** Plasmid pCP13 genes that are differentially expressed in the *virR* mutant compared to the wild type.

| Locus Tag | Log_2_ Fold Change | FDR | Gene^a^ | Product |
| --- | --- | --- | --- | --- |
| PCP01 | -5.34 | 2.88E-07 | *parA* | Soj protein |
| PCP02 | -4.19 | 9.47E-08 | *parB* | ParB protein |
| PCP03 | -5.42 | 5.46E-08 |  | hypothetical protein |
| PCP07 | -6.9 | 3.14E-18 |  | ABC transporter |
| PCP08 | -4.52 | 4.21E-14 |  | hypothetical protein |
| PCP09 | -7.82 | 9.08E-31 |  | hypothetical protein |
| PCP12 | -5.05 | 1.76E-16 |  | hypothetical protein |
| PCP13 | -5.25 | 3.34E-14 |  | hypothetical protein |
| PCP14 | -5.35 | 1.43E-07 |  | hypothetical protein |
| PCP15 | -5.25 | 2.80E-07 | *resP* | resolvase |
| PCP16 | -5.32 | 5.38E-17 |  | hypothetical protein |
| PCP17 | -6.92 | 6.68E-19 | *cpb2* | beta2-toxin |
| PCP18 | -4.24 | 3.72E-08 |  | hypothetical protein |
| PCP19 | -6.17 | 2.75E-12 |  | lineage-specific thermal regulator protein |
| PCP26 | -4.22 | 5.47E-04 |  | hypothetical protein |
| PCP27 | -6.75 | 1.60E-20 |  | hypothetical protein |
| PCP28 | -4.52 | 8.25E-10 |  | hypothetical protein |
| PCP29 | -6.14 | 9.99E-26 |  | hypothetical protein |
| PCP30 | -3.47 | 4.53E-11 |  | hypothetical protein |
| PCP31 | -6.84 | 5.23E-18 |  | hypothetical protein |
| PCP32 | -6.28 | 2.08E-13 |  | resolvase |
| PCP33 | -6.04 | 4.04E-11 |  | hypothetical protein |
| PCP34 | -2.31 | 1.78E-03 |  | hypothetical protein |
| PCP35 | -5.84 | 5.37E-10 |  | DNA primase |
| PCP36 | -3.69 | 9.06E-06 |  | hypothetical protein |
| PCP37 | -4.1 | 1.47E-03 |  | hypothetical protein |
| PCP38 | -6.06 | 9.19E-11 |  | hypothetical protein |
| PCP39 | -6.13 | 1.84E-12 |  | hypothetical protein |
| PCP42 | -3.87 | 3.57E-03 |  | hypothetical protein |
| PCP44 | -4.48 | 9.07E-05 |  | cell wall-binding protein |
| PCP45 | -5.9 | 1.36E-10 |  | hypothetical protein |
| PCP46 | -4.02 | 1.52E-09 |  | conjugal transfer ATP-binding protein TraC |
| PCP47 | -5.75 | 1.30E-09 | *topA* | type I topoisomease |
| PCP49 | -4.74 | 1.63E-05 |  | hypothetical protein |
| PCP50 | -4.33 | 6.47E-09 |  | hypothetical protein |
| PCP51 | -3.66 | 1.72E-07 |  | type IV secretion system protein VirD4 |
| PCP52 | -3.99 | 2.25E-03 |  | hypothetical protein |
| PCP53 | -5.18 | 5.84E-15 |  | hypothetical protein |
| PCP54 | -7.97 | 2.94E-24 |  | hypothetical protein |
| PCP55 | -7.77 | 1.63E-22 |  | Spo0A-like protein |
| PCP56 | -3.77 | 7.34E-03 |  | hypothetical protein |
| PCP57 | -6.26 | 1.94E-20 | *cnaB* | collagen adhesin |
| PCP59 | -6.75 | 5.09E-13 |  | hypothetical protein |
| PCP60 | -6.75 | 1.53E-12 |  | hypothetical protein |
| PCP61 | -4.55 | 1.12E-15 |  | transcription regulator phage-related |
| PCP62 | -4.92 | 8.42E-06 |  | hypothetical protein |
| PCP63 | -3.17 | 7.00E-05 |  | hypothetical protein |

^a^ Fold-change is calculated as the *virR* mutant expression level over the wild type expression level as defined by FDR <0.01 and log_2_ fold change >1. Negative values represent gene expression up-regulated and down-regulated in the *virR* mutant compared to the wild type, respectively.
