# Supplementary material for: RNA-seq analysis of virR and revR mutants of Clostridium perfringens
Source: BMC Genomics. 2016 May 23;17:391. doi: 10.1186/s12864-016-2706-2 (PMC4877802; doi:10.1186/s12864-016-2706-2)
Supplement: Additional file 6: Table S5. — Plasmid pCP13 genes that are differentially expressed in the revR mutant compared to the wild type. (DOCX 13 kb) [file 12864_2016_2706_MOESM6_ESM.docx]

**Table S5:** Plasmid pCP13 genes that are differentially expressed in the *revR* mutant compared to the wild type.

| Locus Tag | Log_2_ Fold Change | FDR | Gene^a^ | Product |
| --- | --- | --- | --- | --- |
| PCP07 | -2.41 | 1.49E-05 |  | ABC transporter |
| PCP08 | -2.02 | 1.01E-03 |  | hypothetical protein |
| PCP09 | -3.41 | 2.20E-08 |  | hypothetical protein |
| PCP29 | -1.73 | 5.25E-03 |  | hypothetical protein |
| PCP53 | -2.17 | 4.04E-04 |  | hypothetical protein |
| PCP57 | -1.84 | 8.12E-04 | *cnaB* | collagen adhesin |
| PCP59 | -2.31 | 8.94E-05 |  | hypothetical protein |

^a^ Fold-change is calculated as the *revR* mutant expression level over the wild type expression level as defined by FDR <0.01 and log_2_ fold change >1. Negative values represent gene expression up-regulated and down-regulated in the *revR* mutant compared to the wild type, respectively.
