# Supplementary material for: RNA-seq analysis of virR and revR mutants of Clostridium perfringens
Source: BMC Genomics. 2016 May 23;17:391. doi: 10.1186/s12864-016-2706-2 (PMC4877802; doi:10.1186/s12864-016-2706-2)
Supplement: Additional file 7: Table S6. — Small RNAs identified from the wild-type depth of coverage plot. (DOCX 17 kb) [file 12864_2016_2706_MOESM7_ESM.docx]

**Table S6:** Small RNAs identified from the wild-type depth of coverage plot.

| SR Number | Transcripts span between | |
| --- | --- | --- |
| 1 | CPE0001 | CPE0002 |
| 2 | CPE0013 | CPE0014 |
| 3^a^ | CPE0041 | CPE0042 |
| 4 | CPE0042 | CPE0043 |
| 5^a^ | CPE0075 | CPE0076 |
| 6 | CPE0083 | CPE0084 |
| 7 | CPE0093 | CPE0094 |
| 8 | CPE0098 | CPE0099 |
| 9^a^ | CPE0102 | CPE0103 |
| 10 | CPE0111 | CPE0112 |
| 11 | CPE1113 | CPE0114 |
| 12^a^ | CPE0128 | CPE0129 |
| 13 | CPE0143 | CPE0144 |
| 14 | CPE0162 | CPE0163 |
| 15 | CPE0174 | CPE0175 |
| 16^a^ | CPE0191 | CPE0192 |
| 17 | CPE0213 | CPE0214 |
| 18 | CPE0238 | CPE0239 |
| 19 | CPE0259 | CPE0260 |
| 20 | CPE0333 | CPE0334 |
| 21^a^ | CPE0334 | CPE0335 |
| 22 | CPE0347 | CPE0348 |
| 23 | CPE0360 | CPE0361 |
| 24 | CPE0369 | CPE0370 |
| 25 | CPE0403 | CPE0404 |
| 26^a^ | CPE0420 | CPE0421 |
| 27 | CPE0454 | CPE0455 |
| 28 | CPE0511 | CPE0512 |
| 29 | CPE0529 | CPE0530 |
| 30^a^ | CPE0534 | CPE0535 |
| 31 | CPE0594 | CPE0595 |
| 32 | CPE0632 | CPE0633 |
| 33^a^ | CPE0670 | CPE0671 |
| 34^a^ | CPE0678 | CPE0679 |
| 35 | CPE0688 | CPE0689 |
| 36^a^ | CPE0693 | CPE0694 |
| 37 | CPE0709 | CPE0710 |
| 38 | CPE0721 | CPE0722 |
| 39 | CPE0738 | CPE0739 |
| 40 | CPE0784 | CPE0785 |
| 41^a^ | CPE0826 | CPE0827 |
| 42 | CPE0870 | CPE0871 |
| 43^a^ | CPE0878 | CPE0879 |
| 44 | CPE0891 | CPE0892 |
| 45 | CPE0910 | CPE0911 |
| 46 | CPE0946 | CPE0947 |
| 47^a^ | CPE0966 | CPE0967 |
| 48^a^ | CPE0997 | CPE0998 |
| 48 | CPE1029 | CPE1030 |
| 50 | CPE1040 | CPE1041 |
| 51^a^ | CPE1166 | CPE1167 |
| 52 | CPE1176 | CPE1177 |
| 53^a^ | CPE1228 | CPE1229 |
| 54 | CPE1233 | CPE1234 |
| 55 | CPE1251 | CPE1252 |
| 56^a^ | CPE1526 | CPE1527 |
| 57^a^ | CPE1603 | CPE1604 |
| 58 | CPE1616 | CPE1617 |
| 59 | CPE1625 | CPE1626 |
| 60^a^ | CPE1661 | CPE1662 |
| 61 | CPE1690 | CPE1691 |
| 62 | CPE1780 | CPE1781 |
| 63 | CPE1786 | CPE1787 |
| 64 | CPE1793 | CPE1794 |
| 65 | CPE1806 | CPE1807 |
| 66^a^ | CPE1886 | CPE1887 |
| 67^a^ | CPE1919 | CPE1920 |
| 68^a^ | CPE1943 | CPE1944 |
| 69 | CPE1977 | CPE1978 |
| 70 | CPE1999 | CPE2000 |
| 71 | CPE2071 | CPE2072 |
| 72 | CPE2075 | CPE2076 |
| 73^a^ | CPE2167 | CPE2168 |
| 74 | CPE2170 | CPE2171 |
| 75 | CPE2171 | CPE2172 |
| 76^a^ | CPE2172 | CPE2173 |
| 77 | CPE2273 | CPE2274 |
| 78^a^ | CPE2276 | CPE2277 |
| 79 | CPE2288 | CPE2289 |
| 80 | CPE2301 | CPE2302 |
| 81^a^ | CPE2302 | CPE2303 |
| 82 | CPE2309 | CPE2310 |
| 83 | CPE2317 | CPE2318 |
| 84 | CPE2321 | CPE2322 |
| 85 | CPE2406 | CPE2407 |
| 86^a^ | CPE2428 | CPE2429 |
| 87 | CPE2522 | CPE2523 |
| 88^a^ | CPE2524 | CPE2525 |
| 89 | CPE2527 | CPE2528 |
| 90^a^ | CPE2541 | CPE2542 |
| 91^a^ | CPE2546 | CPE2547 |
| 92 | CPE2573 | CPE2574 |
| 93 | CPE2632 | CPE2633 |
|  |  |  |

^a^ *in silico* sRNAs by ([Chen *et al*., 2011](#_ENREF_1)).

CHEN, Y., INDURTHI, D. C., JONES, S. W. & PAPOUTSAKIS, E. T. 2011. Small RNAs in the Genus Clostridium. *MBio,* 2.
